# Five-layer systems analysis of *Leishmania* stage differentiation reveals an essential role for protein degradation in parasite development

**DOI:** 10.7554/eLife.111115

**Published:** 2026-08-12

**Authors:** Pascale Pescher, Thibaut Douché, Quentin Giai Gianetto, Karen Druart, Julie Kovářová, Blaise Li, Thomas Cokelaer, K Shanmugha Rajan, Laura Piel, Céline Besse, Anne Boland, Jean-François Deleuze, Mariette Matondo, Michael P Barrett, Shulamit Michaeli, Gerald F Späth

**Affiliations:** 1 Institut Pasteur, Université Paris Cité, INSERM 1347, Unité de Parasitologie moléculaire et Signalisation Paris France; 2 Institut Pasteur, Université Paris Cité, CNRS UAR 2024, Proteomic Platform, Mass Spectrometry for Biology Unit Paris France; 3 Institut Pasteur, Université Paris Cité, Biostatistics and Bioinformatics Hub Paris France; 4 Glasgow Centre for Parasitology, University of Glasgow Glasgow United Kingdom; 5 Weizmann Institute of Science, Department of Chemical and Structural Biology Rehovot Israel; 6 Bar-Ilan University, Mina and Everard Goodman Faculty of Life Sciences and Advanced and Nanotechnology Institutee Ramat-Gan Israel; 7 Université Paris-Saclay, CEA, Centre National de Recherche en Génomique Humaine Evry France; University of Geneva Switzerland; University of Geneva Switzerland

**Keywords:** *Leishmania donovani*, stage differentiation, systems analysis, protein degradation, regulatory networks, Other

## Abstract

Vector-borne, protist parasites have evolved complex developmental programs to adapt to very distinct host environments. How these important pathogens transition between insect and mammalian stages is only poorly understood. Here, we investigated stage differentiation in *Leishmania donovani*, a trypanosomatid parasite with constitutive gene transcription, offering a model to study post-transcriptional regulation. Using a five-layer integrative systems analysis (genome to metabolome), we compared hamster-derived amastigotes and culture-derived promastigotes. Genomic adaptation was excluded as a major driver of differentiation, while differential mRNA turnover emerged as a key mechanism of stage-specific gene expression. Transcriptomic and proteomic comparisons revealed a broad dynamic range of protein abundance changes that correlated poorly with mRNA levels. This discrepancy was linked to (i) altered snoRNA expression and rRNA modifications, indicating stage-specific tuning of translation, and (ii) differential protein degradation, supported by proteomics following proteasome inhibition with lactacystin. Lactacystin impaired amastigote-to-promastigote differentiation, highlighting the importance of proteasomal activity. Overall, our analysis links *Leishmania* development to coordinated post-transcriptional regulatory networks. Our findings provide a powerful new resource for research programs that aim to dissect the emergent properties of regulatory networks and feedback loops underlying *Leishmania* stage differentiation, serving as a blueprint for other vector-borne pathogens that rely on disease-associated developmental transitions.

## Introduction

During their infectious cycle, many protist and fungal pathogens undergo complex developmental transitions that adapt their biology to different host environments, thus ensuring transmission, persistence, or immune evasion. For example, stage differentiation of the malarial parasite *Plasmodium* spp. is tightly controlled at transcriptional levels by the dynamic interplay between epigenetic regulators that remodel the chromatin landscape to accommodate stage-specific transcription factors, such as AP2 DNA-binding family members (Hollin and Le Roch, 2020; Modrzynska et al., 2017). Likewise, the transition from fast-growing tachyzoites to slow-growing bradyzoites during mammalian *Toxoplasma* infection is regulated by the single master TF BFD1 (Waldman et al., 2020). Unlike these apicomplexan parasites, stage differentiation in the trypanosomatid pathogen *Leishmania* spp. is not regulated at the level of transcriptional control, raising the question of how these parasites establish and maintain the various adaptive forms that develop inside their insect and mammalian hosts.

*Leishmania* spp. are protist parasites of humans that represent a global public health problem causing a series of immunopathologies termed the leishmaniases (Alvar et al., 2012; WHO, 2023; Maia et al., 2023; Barbiero et al., 2024). These parasites differentiate into various developmental forms that are adapted for extracellular proliferation inside the sand fly midgut (procyclic promastigotes), transmission from the vector to the mammalian host during uptake of a blood meal (metacyclic promastigotes), and intracellular proliferation inside fully acidified macrophage phagolysosomes (amastigotes) (Handman, 1999). Unlike other eukaryotes, *Leishmania* development is not controlled transcriptionally as protein-coding genes in these parasites lack individual promoters and are constitutively transcribed at all stages involving parasite-specific processes such as polycistronic transcription and trans-splicing (Clayton, 2002; Liang et al., 2003). Despite the largely constitutive gene transcription, different *Leishmania* stages are characterized by striking morphological, biochemical, and metabolic changes (McConville and Ralton, 1997; McConville et al., 2007; Rosenzweig et al., 2008; McConville and Naderer, 2011; Sunter and Gull, 2017; Dandugudumula et al., 2022). These are regulated by a plethora of post-transcriptional mechanisms, including differential mRNA turnover through the binding of various proteins and protein complexes to the 5’ and 3’ untranslated regions (5’ UTRs and 3’ UTRs, respectively) (Trenaman et al., 2019; Boucher et al., 2002; McNicoll et al., 2005; Bringaud et al., 2007; Azizi et al., 2017; Clayton and Estevez, 2011), that can trigger mRNA degradation via deadenylation and decapping (Schwede et al., 2009; Kramer, 2017). Differential gene expression in *Leishmania* can further be regulated at the translational level, e.g., through (i) selective ribosome recruitment by one of the six isoforms of the *Leishmania* cap-binding protein eIF4E that bind to the 5’ UTR, (ii) stabilizing the poly(A) tail via the recruitment of Poly(A)-binding protein 1 (PABP1) to the 3’ UTR promoting translation, or (iii) the differential expression of ribosomal proteins that can increase the landscape of specialized ribosomes (Yoffe et al., 2004; Yoffe et al., 2006; David et al., 2010; Freire et al., 2017; Shrivastava et al., 2021; Assis et al., 2021; Piel et al., 2022; Rodríguez-Almonacid et al., 2023; Gutierrez Guarnizo et al., 2023; Rajan et al., 2024). Finally, differential gene expression in *Leishmania* can also be controlled at the level of protein stability, through stage-specific expression of proteasomal components, autophagy-related genes, or lysosomal proteases (Besteiro et al., 2006; Williams et al., 2006; Besteiro et al., 2007; Williams et al., 2012; Williams et al., 2013; Sakamoto et al., 2021; Casgrain et al., 2016).

While each level of gene expression control has been studied individually in different *Leishmania* species, how these different types of regulation are integrated during *Leishmania* stage differentiation remains to be elucidated. Here, we approached this open question by applying a five-layer, systems-level analysis on the two major life cycle stages of *Leishmania donovani*, i.e., hamster-isolated, bona fide amastigotes and derived promastigotes. Our analysis draws a complex picture of the *Leishmania* differentiation process that emerges from complex, co-regulated genetic networks involving mRNA turnover, protein translation, protein phosphorylation, and protein degradation. Our data reveal *Leishmania* as an interesting model system to analyze phenotypic adaptation in the absence of transcriptional control and to dissociate the role of complex regulatory networks in the development of stable, disease-causing life cycle stages in eukaryotic pathogens.

## Results

### *Leishmania* stage differentiation occurs independently from changes in gene dosage

**Figure 1.**
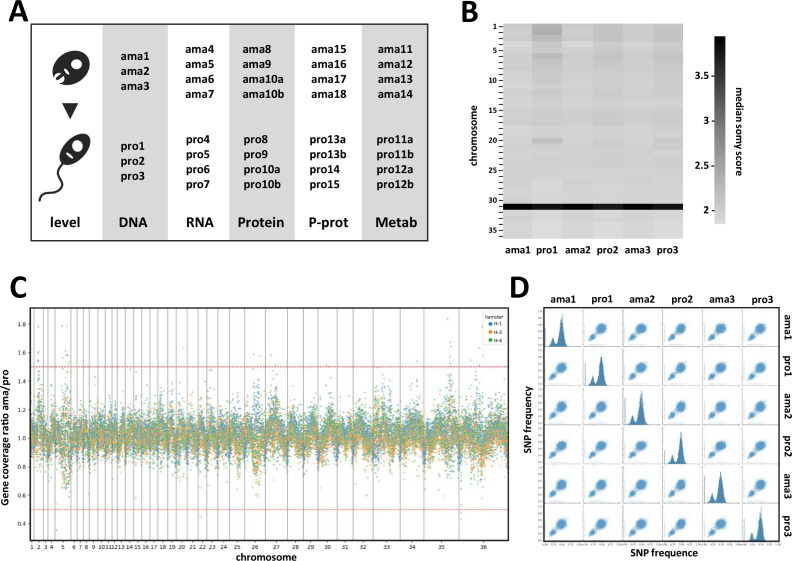
Comparative genomic analysis of *L. donovani* lesion-isolated amastigotes (ama) and derived promastigotes (pro) (N = 3 biological replicates). (**A**) Schematic overview of the parasite samples used for the various systems-level analyses presented in this study. A more detailed overview is shown in Figure 1—source data 1. (**B**–**D**) Genomic analyses. (**B**) Heatmap showing the somy score of three individual differentiation experiments using hamster-isolated amastigotes (ama1, ama2, and ama3) and their corresponding, culture-derived promastigotes analyzed at passage 2 (pro1, pro2, and pro3). Samples and chromosomes are indicated on the x- and y-axis, respectively. The somy scores were calculated as described in Materials and methods and correlate to the gray level according to the shown legend. (**C**) Ratio plot showing the gene coverage ratio ama vs pro (y-axis) for all genes across the 36 chromosomes (x-axis) as calculated based on median read depth normalized by the somy score. Each color represents one individual differentiation experiment using ama isolates from individual hamsters. The red dotted lines indicate the ratio values corresponding to 1.5 (upper line) and 0.5 (lower line), indicating, respectively, gain or loss of one gene copy in the ama samples. Fluctuations between 0.5 and 1.5 were not considered significant. (**D**) Correlation plots representing the individual (histograms on the diagonal) and pairwise (off-diagonal scatterplots) distributions of SNP frequencies for the indicated samples. Of note, only SNPs with a frequency above 10% were plotted. Due to bottleneck events, some low-frequency SNPs appear to be unique in one or the other stage as they are either ‘lost’ (filtered out) or ‘gained’ (passing the 10% cutoff) between the ama and pro samples. The absence of SNPs at 100% is explained by the heterozygous nature of the Ld1S genome. Figure 1—source data 1.Table presenting the details of the samples used in this study.Each *L. donovani* infected hamster is identified by the cage number, as are the hamster-derived amastigotes (ama) and corresponding promastigotes (pro). Figure 1—source data 2.DNA – somy score DNA – gene copy number variation (gCNV). Figure 1—source data 3.DNA – gene copy number variation (gCNV). Figure 1—source data 4.DNA – frequency of SNV.

We carried out an in-depth, systems analysis of independent *L. donovani* amastigote (ama) preparations isolated from infected hamster spleens and their culture-derived promastigotes (pro) at in vitro passage 2, with the aim of revealing mechanisms controlling stage-specific differential expression across five quantifiable information levels, by comparing the genome, transcriptome, proteome, metabolome, and phosphoproteome of the two life cycle stages (Figure 1A, Figure 1—source data 1). We first investigated the possible role of genome instability in stage differentiation, given the very well-documented propensity of *L. donovani* to establish and tolerate chromosome and gene copy number variations in culture and during sand fly or hamster infection (Prieto Barja et al., 2017; Bussotti et al., 2021; Bussotti et al., 2023).

**Figure 1—figure supplement 1.**
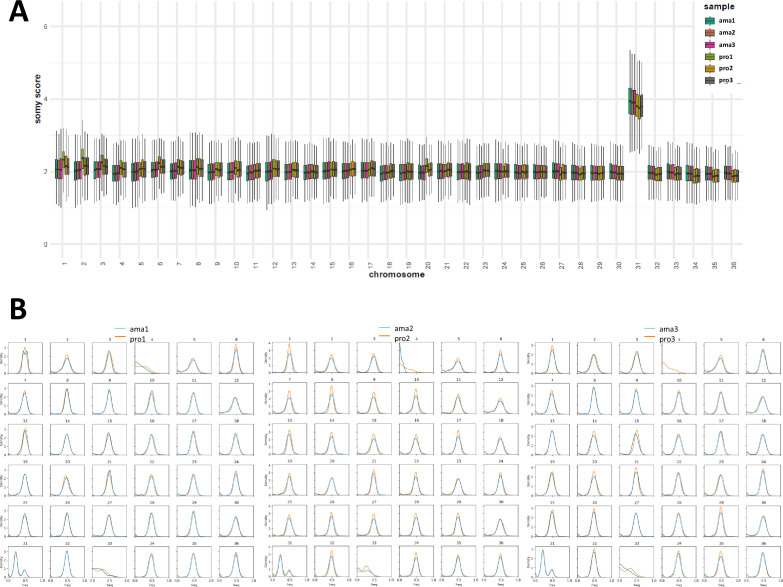
Somy score and SNP analyses. (**A**) Box plots representing the median somy score for all the chromosomes. Each box corresponds to one of three biological replicates for ama and pro as indicated in the graph. Sample identifiers are detailed in the legend of Figure 1A and Figure 1—source data 1. (**B**) Frequency distribution plots representing, for each biological replicate and each chromosome, a kernel density estimate of the distribution of SNP frequencies in the amastigote and promastigote sample.

Applying our computational pipeline termed ‘GIP’ (Späth and Bussotti, 2022) to the genome sequences of three independent pairs of amastigotes and promastigotes confirmed the largely disomic state of the *Leishmania* genome at both stages, except for the known constitutive tetrasomy of chr 31 (Figure 1, Figure 1—figure supplement 1, Figure 1—source data 2). We did not detect any major changes at karyotypic levels following promastigote differentiation, confirming our previous observations that stage differentiation occurs independent of karyotypic adaptation (Prieto Barja et al., 2017; Bussotti et al., 2023). Nevertheless, a clear pattern emerged for promastigotes that showed a slight but reproducible increase in median coverage for chr 1, 2, 3, 4, 6, and 20, suggesting the emergence of mosaic aneuploidy for these chromosomes early during culture adaptation. However, these minor signals are not predictive for the karyotypic changes characteristic of culture-adapted Ld1S parasites (i.e. chr 5, 26, 33, Prieto Barja et al., 2017; Bussotti et al., 2023). Likewise, differentiation of promastigotes was not associated with any major gene copy number variation at early passage 2 (Figure 1C, Figure 1—source data 3). Even though a number of genes showed increased (e.g. Ld1S_300477100 encoding an intraflagellar transport protein) or decreased (e.g. Ld1S_220235700 encoding a hypothetical protein) read depth in individual pro strains, none of these changes converged across all pro samples. In the absence of convergent selection, it is impossible to distinguish if these gene CNVs provide some strain-specific advantage or are merely the result of random genetic drift. Plotting the normalized read depth ratios between amastigotes and promastigotes revealed an intriguing, undulating pattern that was highly reproducible across the three independent experiments and has been previously associated with nascent DNA produced during the replicative S-phase of the cell cycle (Marques et al., 2015; Bussotti et al., 2018; Kloehn et al., 2015). Finally, differentiation did not affect the SNP frequency distribution (Figure 1—figure supplement 1B, Figure 1—source data 4), which resulted in a largely diagonal pattern when plotting amastigote against promastigote SNP frequencies (Figure 1D).

In conclusion, unlike long-term culture adaptation, which drives important karyotypic changes (Prieto Barja et al., 2017; Bussotti et al., 2020) that can affect transcript and protein abundance levels as demonstrated by a recent four-layer systems analysis (Cuypers et al., 2022), in vitro differentiation of splenic amastigotes into promastigotes was not associated with significant changes in chromosome or gene copy number, ruling out gene dosage effects as the source of stage-specific expression differences. Thus, *L. donovani* promastigote differentiation in vitro is independent of genomic adaptation, confirming our previous results obtained with sand fly-derived, promastigote parasites (Bussotti et al., 2021; Dumetz et al., 2017).

### Comparative RNA-seq analysis reveals stage-specific, co-regulated gene clusters that are controlled at post-transcriptional levels

**Figure 2.**
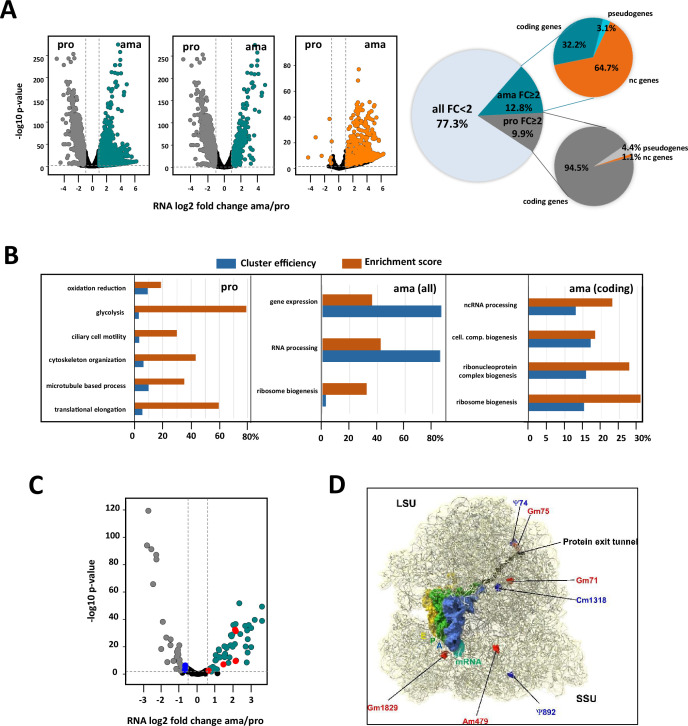
Stage-specific transcript profiling (N = 4 biological replicates). (**A**) Volcano plots (left panels) showing differential transcript abundance between ama and pro samples as assessed by RNA-seq analysis. The dotted lines indicate fold change (FC)=2 (vertical line) and p-value=0.01 (horizontal line). Transcripts with FC<2 or adjusted p-value>0.01 are represented by black dots. Transcripts with significantly increased abundance FC≥2 and adjusted p-value<0.01 in ama and pro are indicated, respectively, in dark cyan and dark gray for all transcripts (left panel) or transcripts of only coding genes (middle panel). Transcripts for non-coding genes with significantly increased abundance FC≥2 and adjusted p-value<0.01 in ama and pro are indicated in the right volcano plot (orange dots). The pie chart (right panel) depicts the percentage of transcripts for each of the categories. (**B**) Gene Ontology (GO) term enrichment analysis of these transcripts for the category ‘biological process’ was performed, and ‘cluster efficiency’ (blue) and ‘enrichment score’ (orange) were plotted for pro (left panel) and ama datasets (all transcripts, middle panel; transcripts for coding genes, right panel). The graphs show some of the most significantly enriched GO terms associated with a Benjamini and Hochberg (BH) p-value<0.05 that were identified using the BiNGO plug-in in Cytoscape. (**C**) Volcano plot corresponding to the differential expression of snoRNA between pro and ama stages. Cyan dots (ama) and gray dots (pro) represent signals with FC≥2 and adj. p-value<0.05. Black dots represent nonsignificant changes. (**D**) Localization of differentially modified rRNA sites on the *Leishmania* ribosome. The RNA modifications are indicated as space filling in the *Leishmania* cryo-EM structure (PDB: 8RXH). The identity of the RNA modification is defined by the label (Ψ, pseudouridine; Gm and Cm refer to guanine and cytosine methylation, respectively; numbers indicate residues). Upregulated and downregulated RNA modifications in amastigotes according to Rajan et al., 2024, are indicated in red and blue, respectively. Figure 2—source data 1.RNA – transcriptomic analysis ama vs pro. Figure 2—source data 2.RNA – snoRNAs.

We next analyzed stage-specific expression changes applying RNA-seq analyses on four amastigote and derived promastigote strains (Figure 2, Figure 2—figure supplement 1). In contrast to the relatively stable genome infrastructure maintained between stages, we observed substantial, stage-specific differences in transcript abundance. From 10,402 detected transcripts in both stages, 6478 showed statistically significant differences (adj. p-value<0.01, base mean reads≥10 reads), including 1234 and 1027 transcripts with a twofold or higher increase in abundance in amastigotes and promastigotes, respectively (Figure 2—source data 1). Considering the lack of promoter-driven expression control of individual, protein-coding genes in *Leishmania* and the absence of stage-specific gene dosage effects (see Figure 1), the observed differences in amastigote and promastigote transcript abundances are most likely attributable to stage-specific mRNA turnover. Interestingly, non-coding (nc) RNAs account for 64.7% of the differentially expressed transcripts at the amastigote stage (Figure 2A).

**Figure 2—figure supplement 1.**
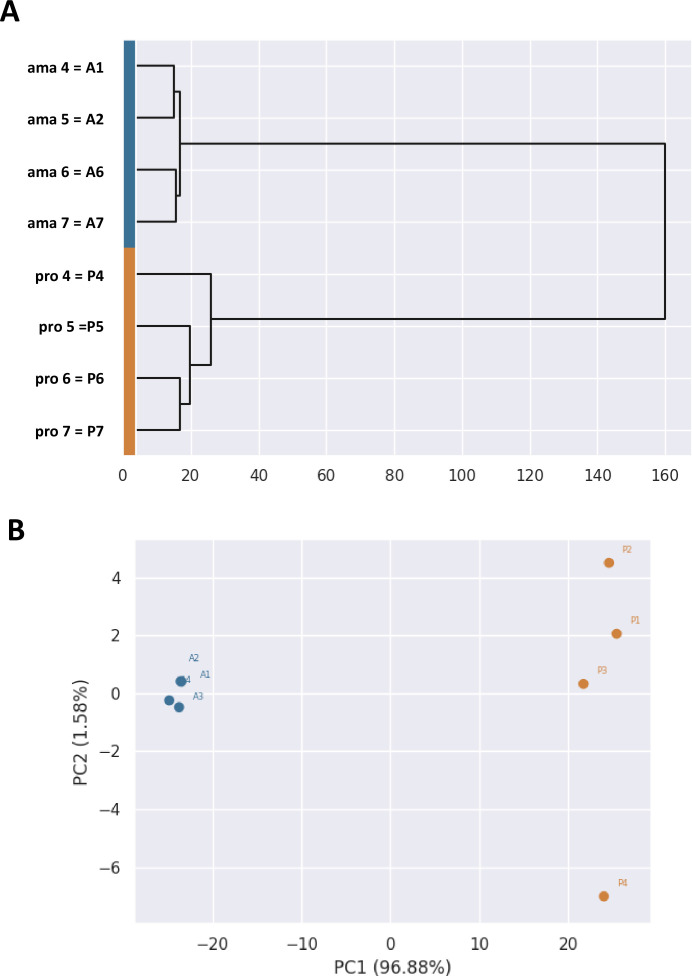
Analysis of RNA-seq data. (**A**) Hierarchical clustering of the samples according to RNA-seq expression profiles. The dendrogram is built using the Ward method. The normed data was log-transformed first and at max 5000 most variable features were selected. (**B**) Principal component analysis. The two main components are represented. Ama and pro samples correspond to blue and orange dots, respectively.

Analyzing the stage-specific RNA-seq datasets identified clusters of co-regulated transcripts that share a common annotation (Figure 2, Figure 2—source data 1). In promastigotes, we observed increased abundance for 66 transcripts annotated for the interconnected Gene Ontology (GO) terms ‘microtubule-based process’, ‘cytoskeleton organization’, and ‘ciliary cell motility’, confirming the post-transcriptional co-regulation of flagellar biosynthesis as previously shown in *L. mexicana* (Fiebig et al., 2015; Figure 2B, left panel, Figure 2—source data 1). Other co-regulated, functional gene sets are defined by the GO terms ‘oxidation reduction’ (46 transcripts), ‘glycolysis’ (15 transcripts), ‘post-transcriptional regulation of gene expression’ (46 transcripts), and ‘translational elongation’ (28 transcripts), further supporting translational control as a regulatory step in stage differentiation.

In amastigotes, a major co-regulated gene cluster is represented by 77 amastin transcripts encoded on 5 different chromosomes that show increased abundance in amastigotes, confirming the stage-specific, post-transcriptional regulation of this gene family as previously described (McNicoll et al., 2005; Wu et al., 2000; Haile et al., 2008; Figure 2—source data 1). Another functional gene cluster showing increased abundance in amastigotes was associated with protein translation, with GO enrichment observed for the terms ‘ribosome biogenesis’ (26 transcripts), ‘ncRNA processing’ (22 transcripts), including 17 transcripts annotated for the GO term ‘rRNA processing’ and 6 transcripts for ‘tRNA processing’ (Figure 2B, middle and right panels, Figure 2—source data 1).

These data suggest ribosome and epi-transcriptomic regulation as possible central components in *Leishmania* stage differentiation. Indeed, analyzing poly-A transcriptome libraries of amastigotes and promastigotes, we observed significant differences in stage-specific expression of 110 pre-snoRNAs (Figure 2C, Figure 2—source data 1). These data fit with the expression of a series of methyl- and pseudouridyl-transferases at this stage (see Figure 3—source data 1). Stage-specific expression of some of these snoRNAs correlated with stage-specific changes previously observed in pseudouridine and 2’-*O*-methylation rRNA modifications (Rajan et al., 2024). The positions of these modifications and the corresponding snoRNAs known to affect ribosome translational activity are indicated on the high-resolution cryo-EM structure of the *Leishmania* ribosome (Figure 2D).

In conclusion, our RNA-seq data reveal a series of functionally related, stage-specific gene clusters whose mRNA abundance is regulated in a coordinated manner at the post-transcriptional level. Whether this co-regulation is governed by stage-specific mRNA decay mechanisms acting on 5’ and 3’ UTRs or by stage-specific mRNA stabilization via RNA-binding proteins or mRNA modification remains to be established.

### Quantitative proteome analysis reveals co-regulated gene clusters that are independent of stage-specific mRNA turnover

**Figure 3.**
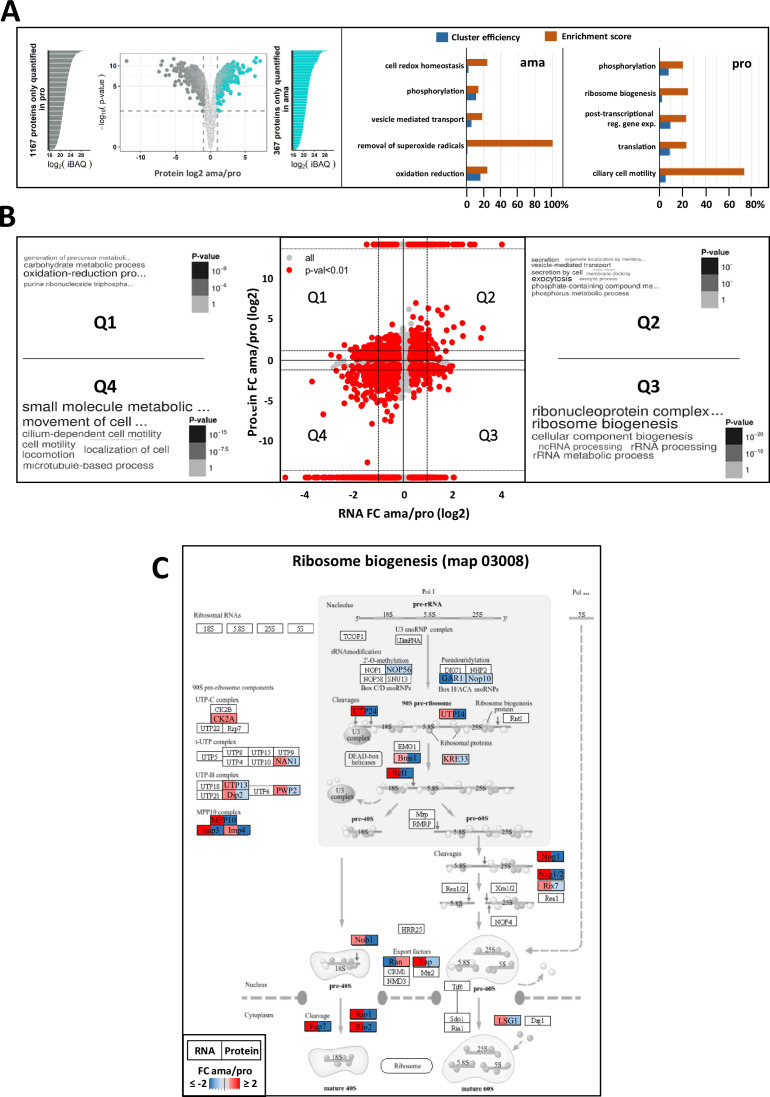
Systems analysis of the stage-specific transcriptome and proteome (N = 4 biological replicates). (**A**) Volcano plot (left panel) showing differential protein abundance between four ama and pro samples as assessed by label-free, quantitative proteomics analysis. The dotted lines indicate fold change (FC)=2 (vertical line) and false discovery rate (FDR)=0.01 (horizontal line). Proteins with FC<2 or FDR>0.01 are represented by light gray dots. Proteins that were reproducibly detected in only one of the two stages (considered unique) are represented by the lateral histograms plotting their relative abundance according to the iBAQ value. These proteins, together with proteins showing differential abundance of FC≥2 and adjusted p-value<0.01, are indicated for ama in dark cyan and for pro in dark gray (see Figure 3—source data 1). Gene Ontology (GO) term enrichment analysis (right panels) for the category ‘biological process’ was performed, and the resulting values for ‘cluster efficiency’ (blue) and ‘enrichment score’ (orange) were plotted for ama (middle panel) and pro (right panel). The graphs show the main GO terms identified using the BiNGO plug-in in Cytoscape and associated with a BH p-value<0.05. (**B**) Double ratio plot showing the log2 FC between ama and pro in transcript (x-axis) and protein (y-axis) abundances. Red dots correspond to changes in both transcript and protein abundance defined either by significant differential abundance at both levels (p-value<0.01) or by significant RNA changes (p-value<0.01) corresponding to proteins detected in only one of the two stages. The dotted lines indicate FC=2. Word cloud enrichment performed with the *L. donovani* LdBPK orthologs is presented for each of the four quadrants, Q1 to Q4, and a detailed GO enrichment analysis is given in Figure 3—source data 2. The font size is proportional to the number of genes per GO term, and the gray scale refers to the p-value calculated for each GO term. (**C**) Kyoto Encyclopedia of Genes and Genomes (KEGG) map for ribosome biogenesis. Each gene associated with expression changes indicated in Q1 to Q4 (see Figure 2C) and annotated with the GO term ‘ribosome biogenesis’ has been projected on the ribosome biogenesis KEGG map. Each gene is represented by a square with the left segment showing FC between ama and pro at transcript and the right segment showing FC at the protein level, with the differential abundance indicated by the color and its intensity (red, increase; blue, decrease). Only genes quantified in both transcriptome and proteome analyses and associated with an adjusted p-value<0.01 were considered. Figure 3—source data 1.Protein – proteomic analysis ama vs pro. Figure 3—source data 2.RNAxProtein – post-transcriptional regulation of gene expression.

We next employed quantitative proteomic analysis to assess how the different co-regulated gene clusters observed in our RNA-seq analyses translate into stage-specific proteomes. Label-free quantitative proteomic analysis of four replicates of amastigotes and derived promastigotes identified over 4000 proteins, including 1987 differentially expressed proteins (adjusted p-value<0.01), and 1534 that were exclusively detected in either ama or pro (Figure 3A, left panel, Figure 3—source data 1). Considering the 822 proteins that are specific or more abundant in amastigotes (fold change≥2), functional enrichment was mainly observed for the GO terms ‘oxidation reduction’ (61 proteins), ‘vesicle-mediated transport’ (23 proteins), and ‘phosphorylation’ (41 proteins) (Figure 3A, middle panel, Figure 3—source data 1). Considering the 1637 proteins that are specific or more abundant in promastigotes (fold change≥2), functional enrichment was observed for the GO terms ‘post-transcriptional regulation of gene expression’ (Marques et al., 2015), ‘translation’ (69 proteins), ‘ribosome biogenesis’ (21 proteins), and ‘ciliary cell motility’ (42 proteins) (Figure 3A, right panel, Figure 3—source data 1).

**Figure 3—figure supplement 1.**
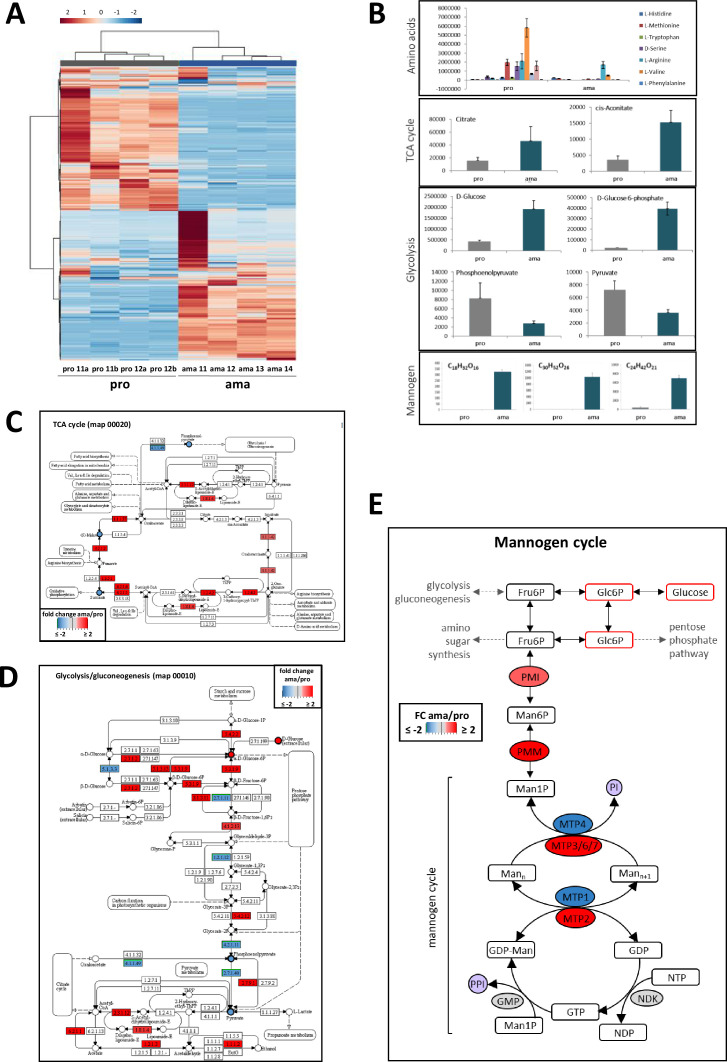
Metabolomics analysis. (**A**) Cluster analysis of metabolites showing differential abundance in ama and pro samples. (**B**) Quantification of amino acids or metabolites involved in glycolysis, TCA cycle, and mannogen cycle. The y-axis represents the intensity for each metabolite as detected by MS. (**C–D**) Correlation between metabolomic and proteomic analyses for TCA cycle (**C**) and glycolysis/gluconeogenesis (**D**). Fold changes in protein abundance (squares) and metabolite abundance (circles) with adjusted p-values of, respectively, <0.01 and <0.05 were projected on the respective Kyoto Encyclopedia of Genes and Genomes (KEGG) maps. The color intensity reflects the fold change value as indicated by the legends in the graphs. (**E**) Representation of the mannogen pathway. Metabolites are represented by a square, with red-lined squares corresponding to the metabolites that accumulate at the ama stage. Proteins are represented by a circle, with fold changes in protein abundance between ama and pro datasets indicated by color and intensity (red, increase; blue, decrease).

The metabolomic analysis of four independent amastigote and promastigote samples (https://doi.org/10.6084/m9.fisshare.310278449, Figure 3—figure supplement 1) supported our proteomic data. Promastigotes showed increased amino acid biosynthetic activity that matched the changes in protein abundance observed for this pathway, while the amastigote metabolome indicates a shift from glycolytic to TCA cycle-dependent energy production that was previously described (McConville et al., 2007; McConville and Naderer, 2011; Saunders et al., 2021; Figure 3—figure supplement 1), with a notable increase in metabolites involved in butanoate metabolism indicative of a shift between carbohydrate use as a primary energy source to utilization of fatty acid oxidation. The amastigote expression profile and metabolome further indicate the stage-specific production of the storage-carbohydrate mannogen, thus corroborating previous observations (Sernee et al., 2019; Figure 3—figure supplement 1).

Surprisingly, only a few enriched GO terms matched between transcriptomics and proteomics datasets, suggesting additional layers of regulation between the transcriptional and translational levels of gene expression. To investigate this aspect on a genome-wide level, we next plotted the simple numerical values we obtained when calculating the ratios of expression changes between ama and pro observed in the transcriptomics and proteomics datasets (Figure 3B, Figure 3—source data 2). Only expression changes were considered that either showed statistically significant differential abundance at both RNA and protein levels (p<0.01) or showed significant RNA changes (p<0.01) with the corresponding protein being detected in only one of the two stages. These latter proteins are identified by signals that were arbitrarily placed at the upper (detected in ama) or the lower (detected in pro) parts of the graph. Whether these proteins just escape detection due to low expression or are truly not expressed remains to be established. The double ratio analysis of the resulting 2349 gene products defined four quadrants: Quadrants 2 (Q2) and 4 (Q4) correspond to 430 and 1042 genes (Figure 3B, Figure 3—source data 2), respectively, for which stage-specific expression changes correlate between protein and RNA levels, suggesting that the abundance of these proteins may be mainly regulated by mRNA turnover. Q2 (mRNA and protein up in ama) includes the GO terms ‘vesicle-mediated transport’ (17 genes) and ‘lipid metabolic process’ (21 genes) (Figure 3B, Figure 3—source data 2), while Q4 (mRNA and protein up in pro) includes the GO term ‘cell motility’ (41 genes) (Figure 3B, Figure 3—source data 2).

Counter-correlations between RNA and protein abundances (p-value<0.01) were observed for 463 and 414 genes, respectively, falling into quadrants Q1 (RNA down and protein up in ama) and Q3 (RNA down and protein up in pro) (Figure 3B, Figure 3—source data 2). The most important difference was observed for 35 genes annotated for the GO term ‘ribosome biogenesis’ (Figure 3C, Figure 3—source data 2). While this GO term defined a positively regulated gene cluster at mRNA level in amastigotes (see Figure 2B), the same genes show reduced expression at this stage at protein level (Figure 3C). This lack of correlation may be explained by stage-specific changes in mRNA translation efficiency.

In conclusion, while many stage-specific changes of protein abundance are strictly regulated by similar changes at mRNA level, others show discrepancies in stage-specific abundances between transcript and protein levels, revealing regulatory mechanisms that act independent of mRNA stability, e.g., at the level of translational control or differential protein stability.

### Stage-specific protein turnover is required for parasite differentiation

**Figure 4.**
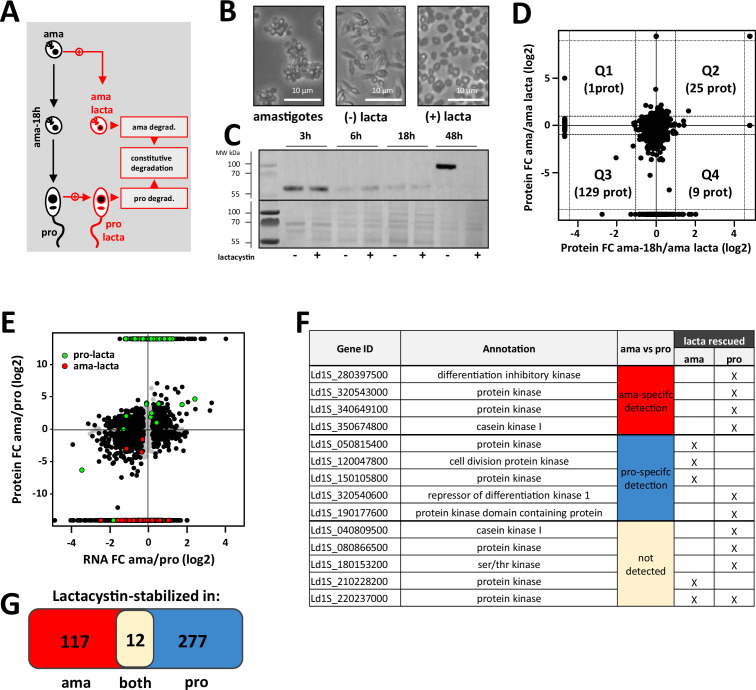
Analysis of stage-specific proteasomal protein turnover (N = 4 biological replicates). (**A**) Overview of experimental workflow. To assess early and late steps of differentiation (left part of the graph in black), comparative, quantitative proteomics analyses were performed on tissue-isolated amastigotes (ama), amastigotes at 18 hr into the transition to promastigotes in culture (ama-18h) and fully differentiated promastigotes after one passage in vitro (pro). To gain insight into stage-specific and constitutive protein degradation (right part of the graph in red), amastigotes and promastigotes were treated with the irreversible inhibitor lactacystin (ama-lacta and pro-lacta). (**B**) Microscopic images of amastigotes at 18 hr in the absence of lactacystin treatment (amastigotes, left), after 48 hr in the absence ((-) lacta) or the presence of the inhibitor ((+) lacta). (**C**) Western blot analysis. Protein extracts were obtained from 2×10^6^ parasites after 3, 6, 18, and 48 hr of differentiation from amastigotes to promastigotes in the presence (+) or the absence (-) of lactacystin. Extracts were separated by electrophoresis, transferred onto PVDF membrane and the presence of PFR2 was revealed using an anti-PFR2 antibody (upper panel). A Coomassie blue stain of the same gel is shown as loading control (lower panel). (**D**) Double ratio plot comparing the log_2_ fold changes (FC) in protein abundance between ama-18hr/ama-lacta (x-axis) and ama/ama-lacta (y-axis). Dashed lines indicate FC = 2. (**E**) Projection of the proteins stabilized in the presence of the inhibitor in ama (ama-lacta, red) and pro (pro-lacta, green) onto the double ratio plot shown in Figure 3B. Note that ama-specific proteins are rescued from degradation in pro-lacta, while pro-specific proteins are rescued from degradation in ama-lacta. (**F**) List of the protein kinases stabilized after lactacystin treatment in pro or ama (columns lacta rescued) and either not detected in the total proteome (column ama vs pro, beige cell) or specifically quantified at the ama (red cell) or the pro (blue cell) stage. (**G**) Venn diagram showing the number of proteins specifically stabilized in ama or pro or stabilized in both stages after lactacystin treatment. Figure 4—source data 1.Protein – global proteomic analysis of lactacystin-treated ama and pro vs untreated parasites. Figure 4—source data 2.Protein – proteins modulated during the first 18 hr of ama to pro differentiation. Figure 4—source data 3.Protein – protein stabilization in ama and pro in the presence of lactacystin. Figure 4—source data 4.List of protein kinases stabilized in the presence of lactacystin.Their corresponding orthologs in *L. donovani* strain LdBPK and *L. mexicana* are indicated, as well as the knockout phenotype as published by Baker et al., 2021. Figure 4—source data 5.PDF file containing original western blot and SDS-PAGE for Figure 4C, indicating the relevant bands and treatments. Figure 4—source data 6.Source images of the western blot and SDS-PAGE presented in Figure 4C.

The discrepancies we observed in a subset of genes between changes in mRNA and protein abundance during the amastigote-to-promastigote transition (see Figure 3B) primed us to investigate the role of proteasomal protein degradation in *Leishmania* differentiation. We chose the highly specific and irreversible proteasome inhibitor lactacystin (Zhang and Lin, 2021; Omura and Crump, 2019) over the typanosomatid-specific, reversible drug candidate LXE408 (Nagle et al., 2020) as the latter’s potent cytotoxicity can confound direct effects on protein turnover with secondary consequences of cell death, limiting its utility for dissecting proteasome function in living parasites. Comparative, quantitative proteomics analyses were performed with (i) spleen-derived amastigotes (ama), (ii) amastigotes after the first 18 hr of differentiation in the absence (control, ama-18h) and the presence of lactacystin (ama-lacta), and (iii) promastigotes in culture without (pro) and after 18 hr of treatment with lactacystin (pro-lacta) (Figure 4A, Figure 4—figure supplement 1, Figure 4—source data 1).

**Figure 4—figure supplement 1.**
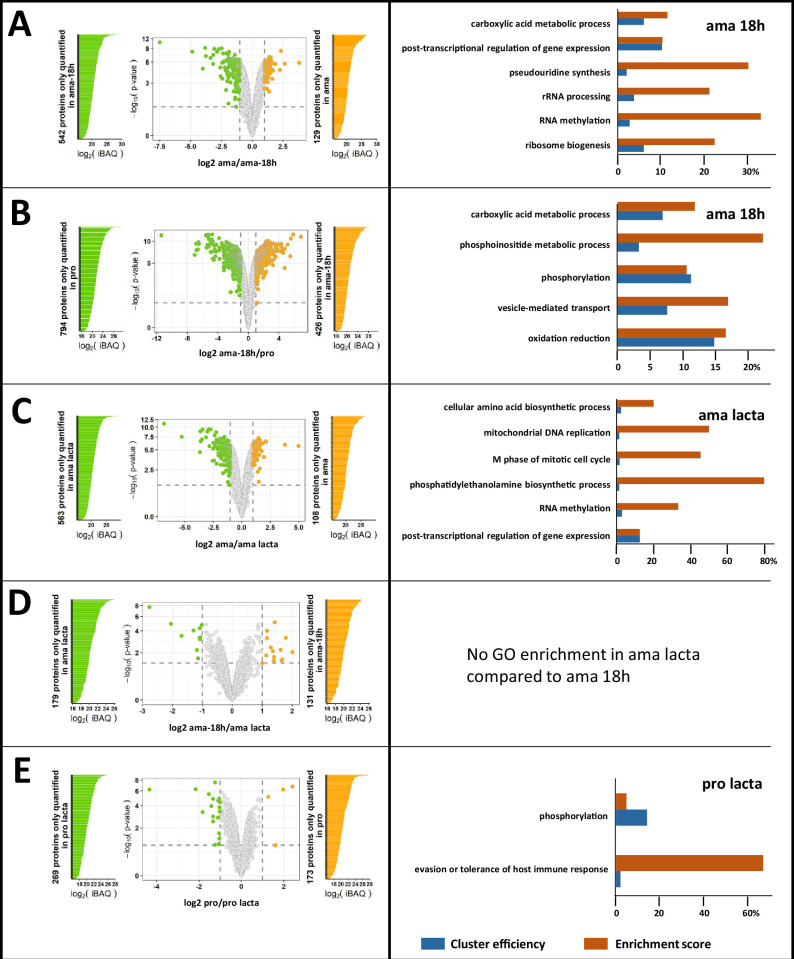
Label-free quantitative proteomic analyses in the presence or absence of lactacystin. (**A–E**) Volcano plots representing changes in protein abundance for the different comparisons (left panels) and Gene Ontology (GO) term enrichment analyses for the indicated sample (right panels). Proteins identified by at least two peptides in at least three out of four biological replicates were considered. For volcano plots, colored dots indicate values with false discovery rate (FDR)<0.01 and fold change (FC)≥2 (see Figure 4—source data 1). The light gray dots indicate nonsignificant expression changes. The colored bars indicate unique protein identifications in one (green) or the other (orange) condition, with relative abundance indicated by the iBAQ value (left panels). Proteins quantified in only one condition or showing a significant increase in abundance (FC≥2 and adjusted p-value<0.01) were used to perform the GO analysis for the category ‘biological process’. The histogram plots show ‘cluster efficiency’ in blue and ‘enrichment score’ in orange (right panels) for a selection of GO terms identified using the BiNGO plug-in in Cytoscape and associated with a BH p-value<0.05. (**A**) ama vs ama-18h; (**B**) ama-18h vs pro; (**C**) ama vs ama-lacta; (**D**) ama-18h vs ama lacta; (**E**) pro vs pro-lacta.

**Figure 4—figure supplement 2.**
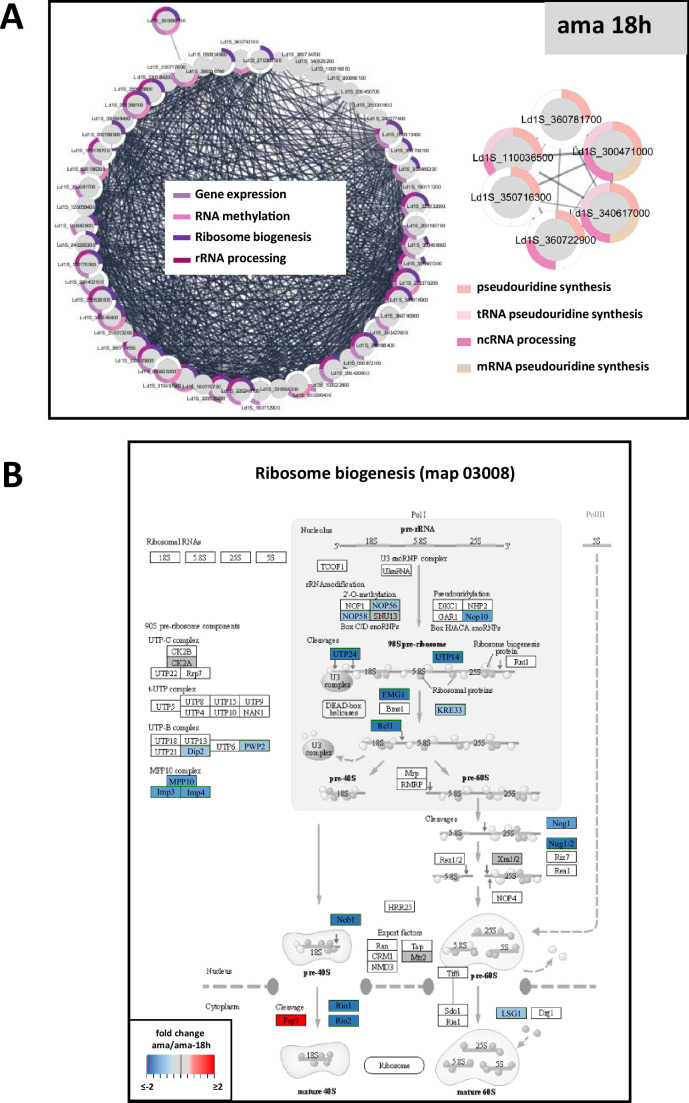
Systems analyses of expression changes. (**A**) Functional network enrichment analysis for proteins showing increased abundance in ama-18h compared to ama. The network was generated with the *L. infantum* orthologs using the STRING plug-in of the Cytoscape software package and considering the full STRING network with a confidence score cutoff of 0.4. The Gene Ontology (GO) terms associated with each protein are represented by the colored segments according to the legend. Only GO terms associated with a p-value<0.05 were considered. (**B**) Kyoto Encyclopedia of Genes and Genomes (KEGG) map for ribosome biogenesis showing changes in abundance protein between ama and ama-18h. Only proteins with an adjusted p-value<0.01 were considered. Differential abundance is indicated by the color and its intensity (red, increase; blue, decrease).

**Figure 4—figure supplement 3.**
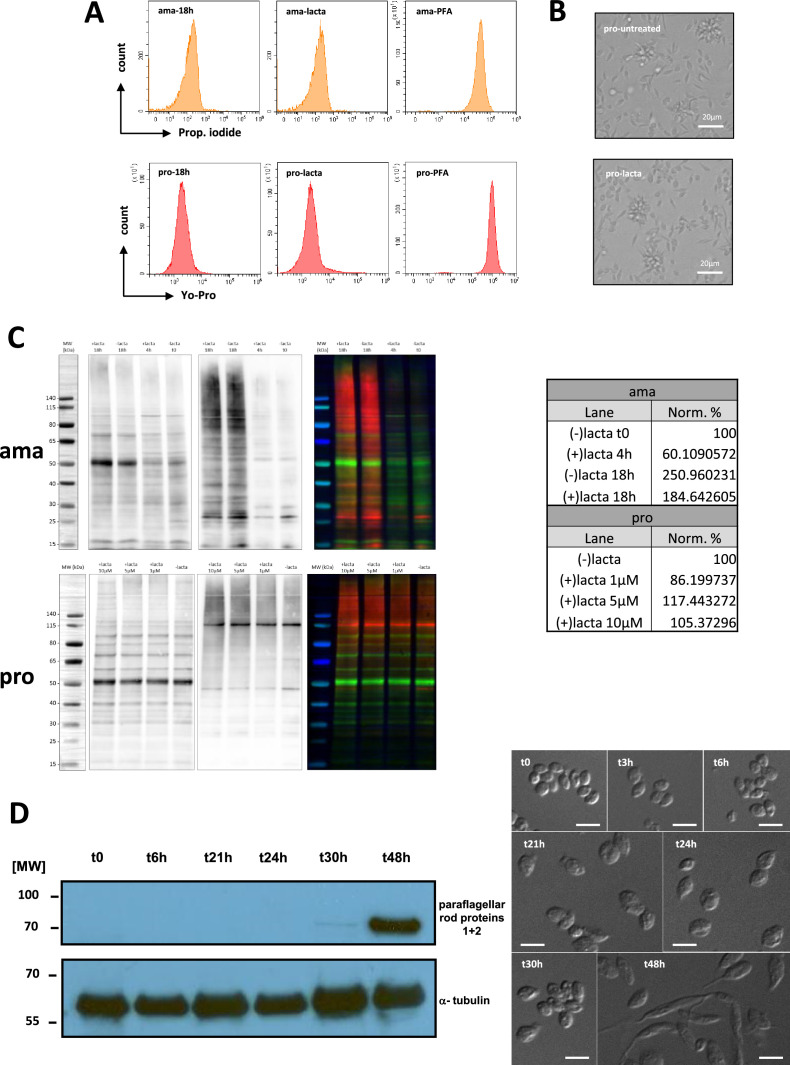
Analyses of lacatcystin-treated parasites. (**A**) Viability assessment of the indicated amastigote (upper panel) and promastigote (lower panel) samples after lactacystin treatment by FACS analysis using propidium iodide or YoPro staining. (**B**) Microscopic images of promastigotes at 18 hr in the absence (upper image) or the presence of 10 µM of lactacystin (lower image). (**C**) Western blot analysis of protein extracts obtained from ama (upper image) and pro (lower image) in the presence or the absence of lactacystin treatment. 10 µg of proteins were labeled with Cy5, separated on NuPAGE 4–12%, and transferred onto PVDF membrane. Membranes were subsequently incubated with a monoclonal antibody against ubiquitin and revealed with HRP-conjugated secondary antibodies. Images of Cy5-labeled proteins (left images), ubiquitinated proteins (middle images), and an overlay of both images (right images, Cy5-labeled proteins in green, ubiquitinated proteins in red) are presented. The table shows the relative quantification after normalization using the Cy5-labeled proteins of untreated samples as a loading control. (**D**) Western blot analysis of protein extracts obtained at the indicated time points after in vitro differentiation from hamster-derived amastigotes to promastigotes. 10 µg of protein were separated on NuPAGE 4–12%, transferred onto PVDF membrane, subsequently incubated with antibodies against paraflagellar rod proteins 1 and 2 and alpha-tubulin, and revealed with HRP-conjugated secondary antibodies (left image). Micrographs showing hamster-derived amastigotes during in vitro differentiation to promastigotes (right image). Parasites were collected at the time points indicated, fixed in 4% PFA, and seeded on poly-L-lysine-treated coverslips. Parasites were observed with a 63× oil immersion objective, and images were processed with AxioVision Rel.4.8 software. Differential interference contrast (DIC) microscopy was performed with Axioplan 2 imaging microscope using a 63× oil immersion objective, Axiovision software, and AxioCam MRm camera (Carl Zeiss). The scale bars correspond to 1 µm. Figure 4—figure supplement 3—source data 1.PDF file containing original western blots presented in Figure 4—figure supplement 3C and D, indicating the relevant bands and treatments. Figure 4—figure supplement 3—source data 2.Source images of the western blots presented in Figure 4—figure supplement 3C and D.

**Figure 4—figure supplement 4.**
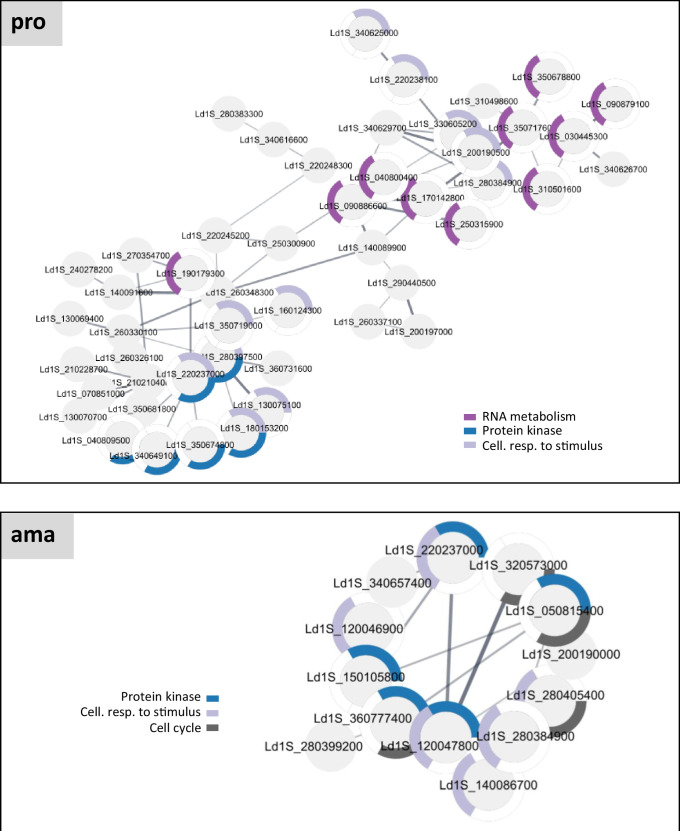
Functional network enrichment analysis for the proteins stabilized in the presence of lactacystin in promastigote (pro) and amastigote (ama) datasets. The network was generated with the *L. infantum* orthologs using the STRING plug-in of the Cytoscape software package and considering the full STRING network with a confidence score cutoff of 0.4. The Gene Ontology (GO) terms associated with each protein are represented by the colored segments according to the legend. Only GO terms associated with a p-value<0.05 were considered.

This dataset allowed us important insight into processes of early differentiation, revealing increased abundance in ama-18h compared to ama for 694 proteins (fold change≥2, adjusted p-value<0.01) (Figure 4—figure supplement 2, Figure 4—source data 2) with GO enrichment observed for the terms ‘ribosome biogenesis’, ‘RNA methylation’, ‘rRNA processing’, ‘gene expression’, and ’post-transcriptional regulation of gene expression’, the latter including six genes encoding for pseudouridine transferases known to modify ribosomal (r) RNA (Figure 4—figure supplements 1 and 2) (Figure 4—figure supplement 2, Figure 4—source data 2). These data further sustain a role of epitranscriptomic regulation and stage-adapted ribosomes as key processes that may initiate *Leishmania* promastigote differentiation, at least in culture (Piel et al., 2022; Rajan et al., 2024; Bussotti et al., 2021).

Lactacystin treatment further uncovered an essential regulatory role of proteasomal protein degradation in *L. donovani* development, which builds on previously published observations (Silva-Jardim et al., 2004). While the presence of the inhibitor neither affected parasite viability nor morphology (Figure 4—figure supplement 3) or promastigote growth (as previously shown by Silva-Jardim et al., 2004), lactacystin treatment abrogated the amastigote-to-promastigote developmental transition as judged by the persistence of an amastigote-like morphology (i.e. oval cell shape, retracted flagellum), and the absence of PFR2 expression characteristic of promastigotes (Figure 4B and C). In contrast, untreated amastigotes spontaneously converted to flagellate promastigotes after 48 hr in culture (Figure 4—figure supplement 3). Proteasome inhibition blocked amastigote-to-promastigote differentiation, without inducing rapid global accumulation of ubiquitinated proteins (Figure 4—figure supplement 3), consistent with a quiescent-like state and low basal ubiquitin-proteasome system activity in amastigotes. After 18 hr, ubiquitination levels remained similar to untreated cells, indicating that protein turnover and ubiquitin accumulation are primarily driven by developmental remodeling rather than acute proteasome inhibition. In promastigotes, the lack of detectable change (Figure 4—figure supplement 3) may also reflect high basal ubiquitination, engagement of compensatory pathways such as autophagy, and/or only partial proteasome inhibition.

Comparing the proteomics signatures of lactacystin-treated samples and untreated controls (Figure 4—figure supplement 1) allowed us to reveal the proteasomal targets, whose rescue correlated with the observed abrogation of differentiation. This analysis is complicated by the fact that amastigotes will not only respond to the 18 hr treatment but also initiate their differentiation into promastigotes during this period, at least in the untreated control sample. To distinguish between changes caused by lactacysin treatment and changes due to the differentiation process, we plotted the ratio of ama/ama-lacta against the ratio ama-18h/ama-lacta, which defined one quadrant (Q3) representing 129 proteins whose increased abundance in ama-lacta was exclusively due to rescue from degradation (Figure 4D, Figure 4—source data 3). Applying STRING and GO analyses on these proteins, some of the most significantly enriched terms included ‘protein kinase’, ‘cellular response to stimulus’, and ‘cell cycle’ (Figure 4—figure supplement 4, Figure 4—source data 3).

Proteomics analysis of lactacystin-treated promastigotes (pro-lacta) revealed the rescue of 289 proteins from degradation when compared to untreated control (pro) (Figure 4—source data 3). Comparison of rescued proteins across both ama and pro stages revealed a series of intriguing findings: First, most of these proteins were rescued in a stage-specific manner, confirming differential protein degradation as an important regulatory component in the development and maintenance of *Leishmania* life cycle stages as suggested by previous reports (Besteiro et al., 2007; Kumar et al., 2007; Pérez-Pertejo et al., 2011; Vince et al., 2011; Khare et al., 2016; Wyllie et al., 2019; Burge et al., 2020; Damianou et al., 2020; Bijlmakers, 2020). Second, just like the proteins rescued from proteasomal degradation in ama-lacta, the pro-lacta data set too showed GO enrichment for the terms ‘protein kinase’ and ‘cellular response to stimulus’, involving distinct sets of proteins (Figure 4—figure supplement 4, Figure 4—source data 3). Thus, *Leishmania* differentiation correlates with the expression of complex signaling networks that are established in a stage-specific manner. Third, when projecting the lactacystin-rescued proteins to the established, stage-specific proteomes (see Figure 3), an intriguing pattern emerged: the majority of proteins that were rescued in ama-lacta turned out to be part of the promastigote proteome dataset (Figure 4E, Figure 4—source data 3). Vice versa, many proteins rescued in pro-lacta were part of the amastigote-specific proteome dataset (Figure 4, Figure 4—source data 3), a pattern that affected 14 protein kinases (Figure 4F). Thus, these proteins seem to be expressed constitutively across both stages, while their steady-state stage-specific abundance is regulated by proteasomal degradation. Surprisingly, 12 proteins (including the protein kinase Ld1S_220237000) were rescued from degradation in both ama-lacta and pro-lacta but were never identified in the proteomes of the corresponding untreated controls, suggesting that they are constitutively degraded in both stages under our experimental conditions (Figure 4G, Figure 4—source data 3).

In conclusion, our results confirm the important role of protein degradation in regulating the *L. donovani* amastigote and promastigote proteomes and identify protein kinases as key targets of stage-specific proteasomal activities.

### Quantitative phosphoproteome analysis reveals *Leishmania* biological networks associated with stage differentiation

**Figure 5.**
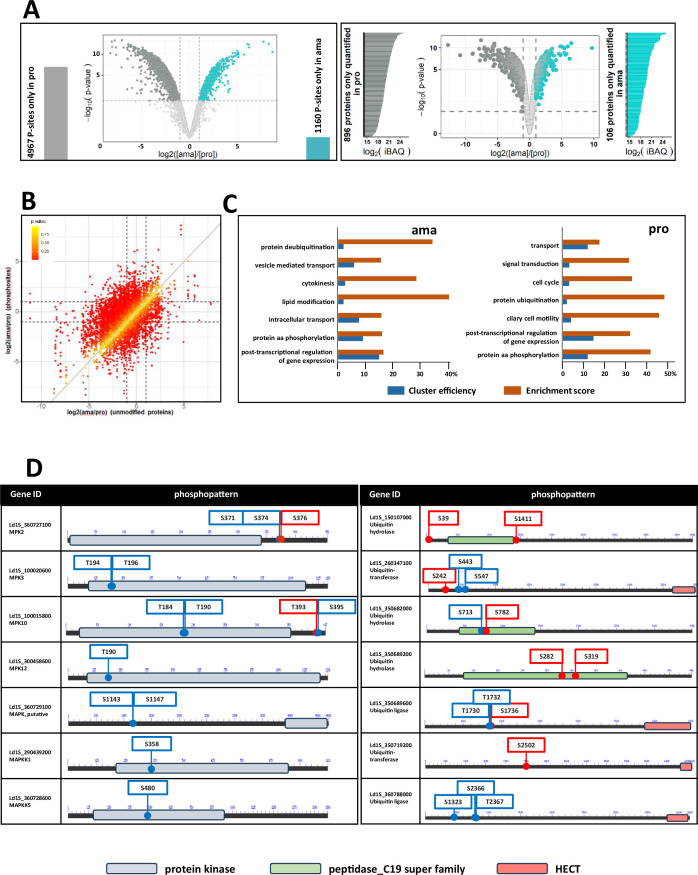
Stage-specific phosphoproteomic profiling (N = 4 biological replicates). (**A**) Volcano plots corresponding to the total proteome (right panel) and phosphoproteome (left panel) analyses. Proteins and phosphosites (P-sites) only identified in one stage are presented at each side of the volcano plots. Cyan dots (ama) and gray dots (pro) represent signals with fold change (FC)≥2 and false discovery rate (FDR)<1%. Light gray dots represent nonsignificant changes. (**B**) Relative phosphorylation change normalized to protein abundance. Ratio plot comparing the log_2_ FC in total protein abundance between ama and pro (x-axis) and phosphosite abundance in ama vs pro (y-axis). Dashed lines indicate an FC = 2. The color intensity of each dot reflects the p-value calculated for the relative phosphorylation change normalized to protein abundance as indicated in the graph. Confidence values were derived as described in Appendix 1. (**C**) Gene Ontology (GO) term enrichment analysis for the category ‘biological process’. Only stage-specific phosphosites were considered (i.e. sites that showed a significant increase in relative phosphorylation normalized to protein abundance, and sites that were only detected in one or the other stage, whether the protein was identified or not in the total proteome analysis). The histogram plots show ‘cluster efficiency’ in blue and ‘enrichment score’ in orange for the GO term enrichment analysis performed with the ama (middle panel) and pro (right panel) datasets. The main GO terms identified using the BiNGO plug-in in Cytoscape and associated with a BH p-value<0.05 are shown. (**D**) Phosphorylation pattern of MAPK and MAPKK proteins (left panel) or ubiquitin hydrolases and transferases (right panel) identified in the relative phosphorylation change normalized to protein abundance. The positions of the phosphorylated amino acid specific to ama (red) and pro (blue) are indicated. Only phosphopeptides identified in the relative phosphorylation change normalized to phosphorylation level (see Figure 5—source data 2) and detected either in one stage or the other or quantified with an FC≥2 were considered for the identification of the phosphosites. Figure 5—source data 1.Phosphoprotein – phosphoproteomic analysis of ama vs pro. Figure 5—source data 2.Phosphoprotein – stage-specific relative phosphorylation changes. Figure 5—source data 3.Phosphoprotein – stage-specific functional networks (STRING).

The investigations above associated the expression of protein kinases (and their degradation) to *Leishmania* stage differentiation (Figure 4F, Figure 4—figure supplement 4), thus confirming previous reports on the importance of phosphotransferase activities in trypanosomatid development (Parsons et al., 2005; Morales et al., 2007; Rotureau et al., 2009; Morales et al., 2010; Potenza et al., 2012; Dacher et al., 2014; Cayla et al., 2014; Jones et al., 2014; Rachidi et al., 2014; Baker et al., 2021; Cayla et al., 2022). Differential turnover of these important signaling proteins by stage-specific proteasomal activities likely represents a regulatory switch controlling parasite development. To further investigate how this putative regulatory switch operates, we screened for stage-specific protein kinase substrates applying label-free, quantitative phosphoproteomic analyses on three independent amastigote and promastigote samples. We identified a total of 2079 phosphopeptides in amastigotes and 7095 phosphopeptides in promastigotes, suggesting a threefold increase in global protein phosphorylation in insect-stage parasites (Figure 5, Figure 5—source data 1). However, increased TiO_2_ phosphopeptide enrichment in a given sample may be due either to increased substrate expression or increased de novo phosphorylation (Figure 5A, Figure 5—figure supplement 1). To distinguish between these two possibilities, we assessed stage-specific relative phosphorylation changes normalized to protein abundance (Figure 5B).

**Figure 5—figure supplement 1.**
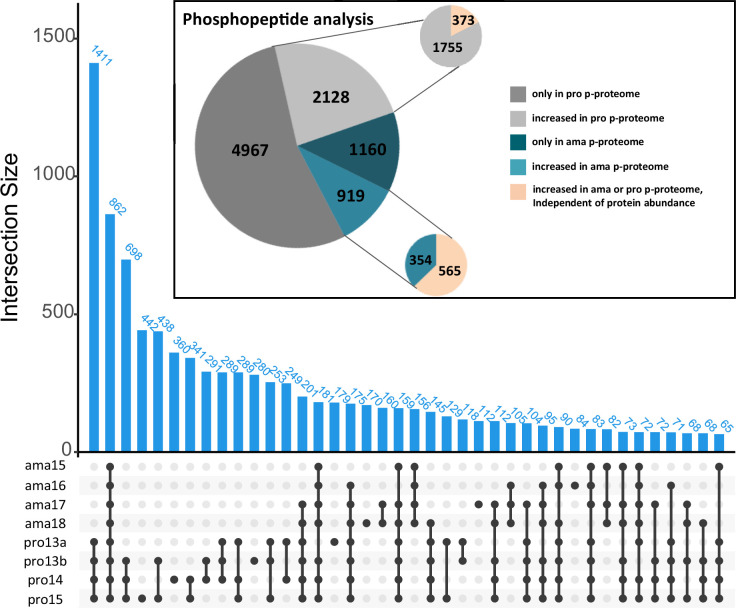
Upset plot representing the number of phosphopeptides (y-axis, blue histograms, numbers are indicated) and the samples in which they were quantified (x-axis). Pie chart of the phosphopeptide analysis showing the distribution of the phosphorylation sites according to the condition in which they were quantified as indicated in the legend of the graph.

**Figure 5—figure supplement 2.**
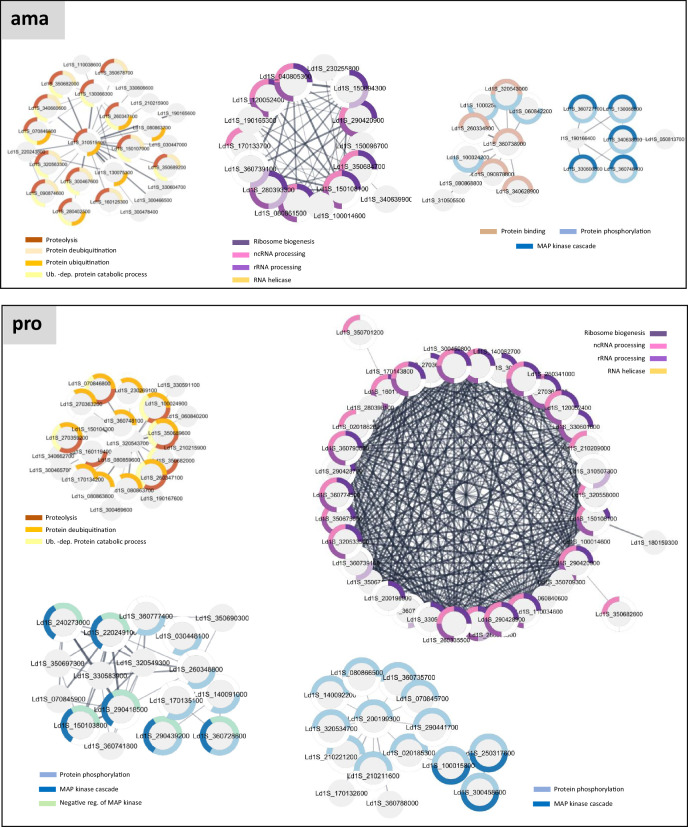
Functional network analysis. Networks for proteins that show unique or increased phosphorylation in ama (upper panel) and in pro (lower panel) datasets were generated with the STRING plug-in of the Cytoscape software package using *L. infantum* orthologs and considering the full STRING network with a confidence score cutoff of 0.4. The nodes represent phosphoproteins associated with one or more Gene Ontology (GO) terms (listed in the graph), with the respective gene identifier indicated. The GO terms associated with the proteins are represented by the colored segments around the nodes according to the legend shown in the graph. Only GO terms associated with a false discovery rate (FDR)<0.05 were considered.

We identified 877 phosphoproteins in amastigote samples carrying 2079 phosphosites of which 55.8% (1160) were uniquely identified at this stage, while 27.2% (565) showed a significant change in normalized phosphorylation levels compared to promastigotes (Figure 5—figure supplement 1, Figure 5—source data 2). Conversely, 2054 phosphoproteins with 7095 phosphorylation sites were identified in promastigote samples, of which 70% (4967) were exclusively detected at this stage, while 5.2% (373) presented a significant change in normalized phosphorylation levels compared to amastigotes (Kloehn et al., 2015; Figure 5—figure supplement 1, Figure 5—source data 2). These data reveal a surprising level of stage-specific phosphorylation in promastigotes, which may reflect their increased biosynthetic and proliferative activities compared to amastigotes, or may be a consequence of culture adaptation.

We next investigated the pathways regulated by phosphotransferase activities analyzing the phosphoproteomes of amastigotes and promastigotes for enriched biological processes (Figure 5C, Figure 5—source data 2). Stage-specific enrichment was observed in the pro dataset for ‘ciliary cell motility’, indicating that flagellar biogenesis and activity are not only regulated by increased protein abundance, but also by increased phosphorylation of flagellar proteins. Surprisingly, in both the ama and pro datasets, similar functions were enriched, including cell division (e.g. ‘cytokinesis’, ‘cell cycle’), signaling (e.g. ‘protein aa phosphorylation’, ‘signal transduction’), or post-transcriptional regulation of gene expression. Thus, aside from stage-specific protein turnover (see above, Figure 4), differential phosphorylation represents yet another layer of regulation that contributes to the establishment of biological networks in *Leishmania* that share similar function in both ama and pro, but whose components are stage-specifically regulated at post-translational levels.

Analyzing stage-enriched, functional networks in further detail using the STRING application of the Cytoscape software package revealed a series of potential regulatory interactions (Figure 5—figure supplement 2, Figure 5—source data 3): A first group of protein kinase substrates is represented by protein kinases themselves with more than 30 and 100 members of this large protein family identified in the ama and pro phosphoproteomes, respectively (see Figure 5D for phosphorylation pattern of some members of the MAP kinase pathway, Figure 5—source data 2). An additional group of protein kinase substrates in both amastigote and promastigote phosphoproteomes includes stage-specific phosphatases (18 in ama and 55 in pro) that can counteract protein kinase activities and even deactivate signaling cascades by dephosphorylation of protein kinase substrates (Figure 5—figure supplement 2, Figure 5—source data 2). Furthermore, the stage-specific phosphoproteomes showed enrichment in functional networks linked to ribosomal biogenesis and RNA processing, suggesting that protein kinases may control the establishment of stage-specific ribosomes. Finally, the identification of networks linked to proteasomal protein degradation in both ama and pro datasets not only further sustains the role of protein turnover in *Leishmania* stage differentiation, but uncovers a possible feedback loop between proteasomal activities controlling stage-specific degradation of protein kinases (see Figure 4), which, in turn, may control stage-specific ubiquitin ligases and deubiquitinating enzymes via differential phosphorylation (see Figure 5D for phosphorylation pattern of selected members of the ubiquitin proteasomal system).

## Discussion

By applying a five-layer systems analysis to spleen-derived bona fide amastigotes and culture-derived promastigotes, we identified a series of functionally related gene clusters that are co-regulated during *L. donovani* stage differentiation. This co-regulation occurs at multiple levels, including transcript stability, proteasomal protein turnover, and protein phosphorylation. Our study underscores the complex molecular architecture of the *Leishmania* developmental process and provides novel insights into possible regulatory feedback loops that may coordinate stage-specific transitions. These results raise important questions regarding the integration of these regulatory networks during differentiation, and their contributions to environmental sensing and adaptive evolution. In the following, we discuss these putative networks in the context of the current literature and propose experimental strategies for their downstream, mechanistic analyses.

**Figure 6.**
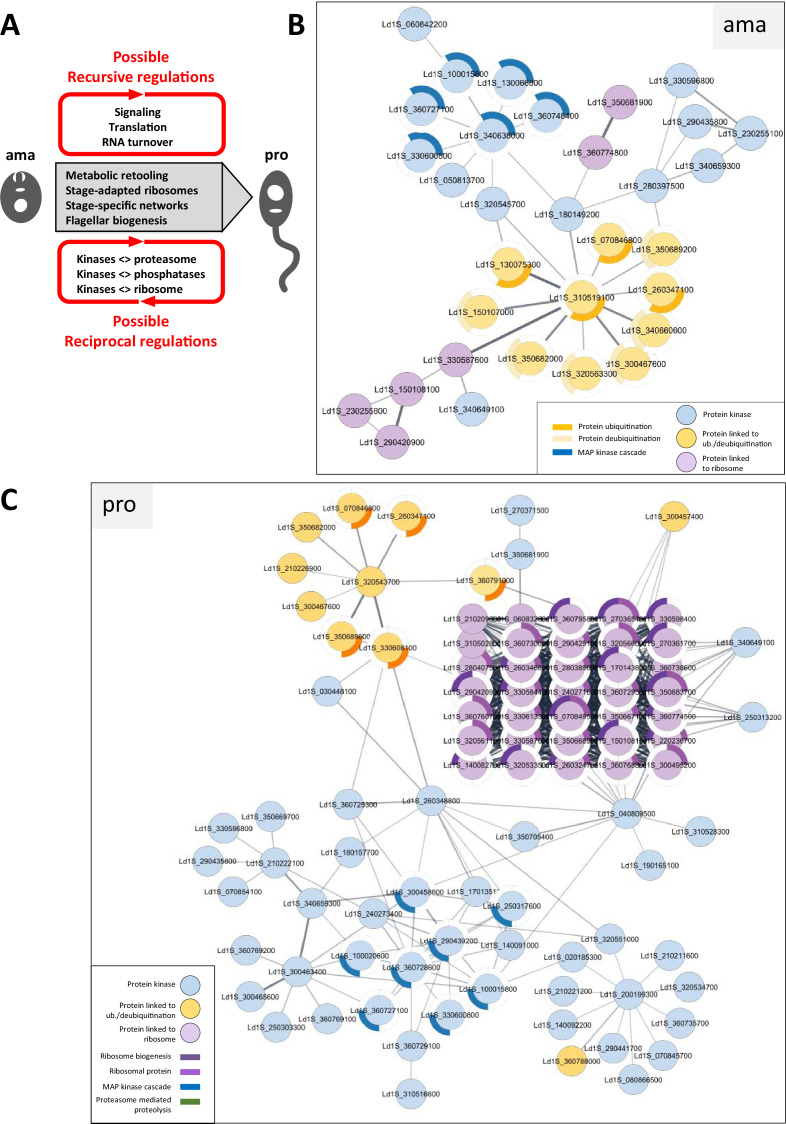
Network analyses. (**A**) Model of *Leishmania* gene expression regulation during stage differentiation. (**B–C**) Network analysis. Networks, restricted to the protein kinases (blue nodes), proteins implicated in ribosome biogenesis and ribosomal proteins (violet nodes), and proteins linked to ubiquitination/deubiquitination (orange nodes), identified based on normalized phosphorylation levels conducted in ama (**B**) or pro (**C**), were generated with the STRING plug-in of the Cytoscape software package using *L. infantum* orthologs and full STRING network with a confidence score cutoff of 0.4. Each node represents a phosphoprotein with the respective gene identifier indicated. Gene Ontology (GO) terms associated with the proteins are represented by the colored segments around the nodes according to the legend shown in the graph. Only GO terms associated with a p-value<0.05 were considered. Figure 6—source data 1.Phosphoprotein – stage-specific interaction networks for selected phosphoproteins (STRING).

A first series of possible regulatory networks proposed by our results could rely on recursive or self-controlling interactions, where components of a given pathway are regulated by the pathway itself (Figure 6A). In promastigotes, for example, a co-regulated, functional gene cluster showing stage-specific increase in transcript abundance was defined by the GO term ‘post-transcriptional regulation of gene expression’. In the absence of stage-specific gene dosage changes (Figure 1) and the lack of transcriptional regulation of gene expression in *Leishmania* (Grünebast et al., 2025), the stage-specific expression changes of these transcripts are thus likely regulated itself at the post-transcriptional level. Such auto-regulatory feedback is common for RNA-binding proteins (RBPs), which play a central role in controlling mRNA stability, often targeting their own transcript (Müller-McNicoll et al., 2019). This helps keep protein levels stable (negative feedback) or create on/off switches for developmental processes (positive feedback) (Perrimon et al., 2012). In *T. brucei,* for example, the stage-specific expression of RBPs, such as ZC3H22 or RBP9, is regulated at post-transcriptional levels (Erben et al., 2021), while other RBPs, such as TbZFP3 or RBP10, can control developmental transitions by stabilizing cohorts of mRNAs, thus defining developmental regulons (Walrad et al., 2012; Mugo and Clayton, 2017). In *Leishmania*, RBPs are likely to participate in comparable regulatory networks. These can be investigated through methods such as crosslinking and immunoprecipitation to map RBP binding to 3’ UTRs, computational approaches to identify conserved regulatory sequence motifs, and loss-of-function analyses targeting either trans-acting factors (RBPs and their interaction partners) or cis-acting elements (e.g. RBP-binding sites within 3’ UTRs).

Our data propose a similar recursive feedback loop that seems to act on the level of protein translation, which was revealed by a paradox: while amastigotes showed increased abundance for 38 transcripts annotated for the GO term ‘ribosome biogenesis’ (Figure 2, Figure 2—source data 1), the corresponding proteins showed reduced abundance in amastigotes compared with promastigotes (Figure 3C). This dissociation between RNA and protein abundances indicates that ribosomal components themselves may be regulated at translational levels. Increased ribosomal protein abundance in promastigotes could satisfy the requirement of increased translation capacity at this fast-growing stage (Piel et al., 2022; Avila et al., 2018). In contrast, increased mRNA abundance of ribosomal components in amastigotes may indicate stage-specific mechanisms of mRNA storage (Fritz et al., 2015) that may allow to jump-start promastigote differentiation and accelerated growth following exposure to low temperature and neutral pH. This possibility is supported by our proteomics investigations of the early differentiation process, revealing ribosomal biogenesis as one of the first pathways induced over the initial 18 hr during the amastigote-to-promastigote developmental transition (Figure 4—figure supplement 2).

Differentiation to promastigotes was associated not only with quantitative changes of ribosomal components as indicated by co-regulated expression of ribosomal proteins and translation factors (see Figure 4—source data 2), but also seems to affect ribosome structure itself as indicated by stage-specific expression changes of gene sets enriched in the GO terms ‘RNA methylation’ (eight methyltransferases, including the rRNA methyltransferases Ld1S_350672300 and Ld1S_350694000) and ‘pseudouridine synthesis’ (six pseudouridine synthases and the H/ACA snoRNP Nop10, Ld1S_360722900) (Figure 4—figure supplement 1, Figure 4—source data 2). The differential expression of snoRNAs (see Figures 2C and 3, Figure 3—source data 2) and the developmentally regulated changes in rRNA modification that can affect ribosome activity (Rajan et al., 2024; Figure 2D) indeed support the existence of such stage-specific ribosomes. These results are in line with our recent reports providing indication for specialized ribosomes in *Leishmania* adaptation, in which we correlated changes in rRNA pseudouridylation to *L. donovani* fitness gain in culture (Piel et al., 2022; Bussotti et al., 2021). Cryo-EM analysis of purified ribosomes from amastigotes and derived promastigotes, combined with targeted deletion of individual snoRNA genes, will be critical to uncover stage-specific structural and functional adaptations of such stage-specific ribosomes.

Finally, a third possible recursive feedback loop seems to act at the post-translational level involving members of various protein kinase (PK) families, which not only showed stage-specific expression, but also represented a main class of phosphorylation substrates themselves. This interaction may reflect stage-specific signaling cascades, where downstream kinases are activated through phosphorylation by upstream kinases, such as exemplified by the MAP kinase pathway (Cayla et al., 2022; Cargnello and Roux, 2011). Indeed, 11 members of the MAP kinase family showed stage-specific changes in their phosphorylation pattern (Figure 5D), 9 of which were only detected in the promastigote phosphoproteome and were previously associated with this stage, being implicated in flagellar biogenesis (MPK9 and MPK3) or metacyclogenesis (MPK4) (Dacher et al., 2014; Baker et al., 2021; Erdmann et al., 2006). In contrast, the two MAP kinases MPK2 and MPK10 – previously linked to amastigote differentiation and virulence (Cayla et al., 2014; Baker et al., 2021) – showed an amastigote-specific increase in protein abundance and relative phosphorylation change for defined phosphorylation sites. In *L. mexicana*, such a recursive PK feedback loop has been identified between the interacting PKs LmxMKK and LmxMPK3, which cross-phosphorylate each other to regulate flagellar length (Erdmann et al., 2006). To further dissociate the complex regulatory relationships between parasite PKs, protein-protein interaction maps should be established (e.g. co-IP, proximity labeling), combined with in vitro kinase assays to reveal direct kinase-substrate relationships.

Most PKs have pleiotropic effects across biological systems, due to their ability to phosphorylate a wide range of functionally unrelated substrates (Johnson et al., 2023). Our analysis of relative phosphorylation changes normalized to protein abundance confirms such pleiotropic function in *Leishmania* and suggests parasite PKs at the center of a second form of possible feedback loop defined by reciprocal interactions, where components of two or more regulatory pathways may cross-control each other (Figure 6A). For example, as judged by changes in phosphorylation abundance, it seems that stage-specific PK activities act on a series of phosphatases, which in return may modify kinase activities by dephosphorylation (Figure 5, Figure 5—source data 3). Such reciprocal regulation between both enzyme families is well established across many eukaryotic systems, e.g., between mammalian ERK1/2 and the MAPK phosphatase DUSP6/MKP-3 (Ekerot et al., 2008), or yeast CDK1 and the phosphatase Cdc25, controlling mitotic entry (Hoffmann et al., 1993). In addition, 47 differentially phosphorylated proteins were associated with ‘Translation’, some of which have been linked in *Leishmania* to translational control, cell cycle regulation, or infectivity (Shrivastava et al., 2021; Zinoviev et al., 2011; Shrivastava et al., 2019; Tupperwar et al., 2019; Tupperwar et al., 2021; Baron et al., 2024). This opens the possibility of yet another reciprocal feedback loop, where phosphorylation of ribosomal components could fine-tune translational control, which in turn may affect the expression of PKs themselves (Baird and Wek, 2012; Sloan et al., 2017; Imami et al., 2018; Merrick and Pavitt, 2018; Piazzi et al., 2019; Jungers et al., 2020; Janin et al., 2020; Bohlen et al., 2021; Khoshnevis et al., 2022).

Finally, our results suggest the possibility of a third reciprocal feedback loop between PKs and proteins of the proteasomal system that may govern *Leishmania* stage transitions. Previous genetic analyses demonstrated an essential role for various components of this system in promastigote-to-amastigote transition and intracellular survival, including *L. mexicana* ubiquitin-conjugating (E2) enzymes (UBC1/CDC34, UBC2, and UEV1), the E3 ubiquitin ligase HECT2, or various deubiquitinases (e.g. DUBs 4, 7, 13) (Burge et al., 2020; Damianou et al., 2020). By applying the irreversible proteasome inhibitor lactacystin, we extended this role to the reverse process – amastigote-to-promastigote differentiation – demonstrating that proteasomal activities are critical for both directions of the developmental cycle (Figure 4). The constitutive expression of many proteasomal components across both amastigote and promastigote stages supports a role of phosphorylation in regulating stage-specific proteasomal activities, considering that phosphorylation of DUBs has been linked to changes in their stability, localization, specificity, or catabolic activity (Das et al., 2020; Wang et al., 2021). We indeed identified 11 DUBs and 10 ubiquitin ligases/transferases showing stage-specific phosphorylation at specific residues (Figure 5, Figure 5—source data 3). This correlated with the stage-specific proteasomal degradation of 14 PKs (Figure 4F, Figure 4—source data 3), including two previously implicated in *Leishmania* in vivo fitness (repressor of differentiation kinase 1, Ld1S_320540600; differentiation inhibitory kinase, Ld1S_280397500, see Figure 4—source data 4; Baker et al., 2021). These findings support the existence of a possible kinase/proteasome reciprocal feedback loop in early parasite development. The experimental validation of such regulatory interactions will require in-depth biochemical, molecular, and systems biology approaches involving global kinase-substrate mapping (Johnson et al., 2023), in vitro reconstitution assays using recombinant proteins to demonstrate reciprocal kinase and proteolytic activities, and genetic perturbation tests to reveal the biological impact of the regulatory interaction.

The complexity of regulatory interactions between PKs and the proteasomal pathway is further increased by showing that substrate phosphorylation can mediate subsequent ubiquitination and degradation, while ubiquitination of MAP kinase family members can regulate kinase activity and localization rather than promote degradation (Hunter, 2007b; Nguyen et al., 2013; Barbour et al., 2023). Future experiments simultaneously analyzing changes in both phosphoproteome and ubiquitome during *Leishmania* stage differentiation and making precision mutants that lack the ubiquitination or phosphorylation sites may help elucidate this complex regulatory interaction and identify individual proteins and their PTMs that drive parasite development.

In conclusion, our data represent an important novel resource to experimentally assess the regulatory landscape of *Leishmania* stage development. We propose genetic feedback control as a central mechanism in parasite differentiation, with stage-specific interactions between genes and their products likely adapting the parasite phenotype to the environmental changes encountered inside its vertebrate and invertebrate hosts. In contrast to the major phenotypic shift observed between mammalian-stage amastigotes and insect-stage promastigotes, each developmental stage is stably maintained within its respective host even in the wake of environmental fluctuations. Such phenotypic robustness is known to rely on highly interconnected, stage-specific regulatory networks (Wanddington, 1959), which in the case of *Leishmania* could implicate recursive and reciprocal genetic interactions between protein translation, protein degradation, and protein phosphorylation (Figure 6B and C, Figure 6—source data 1). Our results define *Leishmania* differentiation as an excellent model system to study how alterations between phenotypic plasticity (i.e. developmental transitions) and robustness (stable maintenance of stages) shape microbial fitness, and provide a powerful experimental framework for future studies on the emergence of stable, adaptive biological traits through feedback regulation.

## Materials and methods

**Key resources table:** 

Reagent type (species) or resource	Designation	Source or reference	Identifiers	Additional information
Strain, strain background (*Leishmania donovani* 1S2D)	*L. donovani*	Originally obtained from Henry Murray, Weill Cornell Medical College, New York, USA	Strain 1S2D MHOM/SD/62/1S-CL2D	Maintained in hamsters (*Mesocricetus auratus*)
Strain, strain background (*L. donovani* amastigotes)	Amastigotes	This paper		Prepared from hamster infected spleens
Strain, strain background (*L. donovani* promastigotes)	Promastigotes	This paper		Derived from splenic amastigotes
Antibody	Mouse monoclonal antibody anti-PFR 1+2 (clone L13D6)	Philippe Bastin, Institut Pasteur, Paris		WB (1:50)
Antibody	Mouse monoclonal antibody anti-tubulin alpha (clone B-5-1-2)	Sigma	T6074	WB (1:25,000)
Antibody	Mouse monoclonal antibody anti-ubiquitin (clone p4d1)	Abcam	AB303664	WB (1:1000)
Antibody	Goat secondary antibody anti-mouse IgG-HRP conjugated	Invitrogen	32230	WB (1:10,000)
Antibody	Goat secondary antibody anti-rabbit IgG-HRP conjugated	Invitrogen	31462	WB (1:10,000)
Commercial assay or kit	DNeasy Blood and Tissue Kit	QIAGEN	69504	
Commercial assay or kit	Nucleospin RNA plus	Macherey Nagel	740984	
Commercial assay or kit	RCDC Protein assay Kit II	Bio-Rad	5000122	
Commercial assay or kit	Quick Stain Kit	Cytiva	RPN4000	
Commercial assay or kit	rDNase set	Macherey Nagel	740963	
Commercial assay or kit	SuperSignal West Pico PLUS Kit	Thermo Scientific	34577	
Commercial assay or kit	ECL prime western blotting detection reagent	Cytiva	RPN2232	
Commercial assay or kit	Quant-IT kits	Invitrogen	Q33267 (DNA) Q10213 (RNA)	
Commercial assay or kit	TruSeq DNA PCR-Free Library Preparation Kit	Illumina	20015963	
Commercial assay or kit	Illumina Stranded mRNA Prep	Illumina	20040534	
Chemical compound, drug	YO-PRO	Invitrogen	Y3306	
Chemical compound, drug	Propidium iodide	Sigma-Aldrich	P4864	
Chemical compound, drug	Lactacystin	Sigma-Aldrich	L6785	
Chemical compound, drug	Protease inhibitor cocktail cOmplete	Roche	11836170001	
Chemical compound, drug	Phosphatase inhibitor cocktail PhosStop	Roche	04906845001	
Software, algorithm	Sequana	Cokelaer et al., 2017		
Software, algorithm	Sequana RNA-seq	doi: https://doi.org/10.5281/zenodo.19456198		
Software, algorithm	GIP	Späth and Bussotti, 2022		
Software, algorithm	Cytoscape	Shannon et al., 2003		
Software, algorithm	UCSF Chimera-X	Pettersen et al., 2021		

### Animals

Twenty-five female Golden Syrian hamsters (*Mesocricetus auratus* RjHan:AURA, weighing between 50 and 60 g) were purchased from Janvier Laboratories. All animals were handled under specific, pathogen-free conditions in biohazard level 3 animal facilities (A3) accredited by the French Ministry of Agriculture for performing experiments on live rodents (agreement A75-15-01).

### Parasites and culture

*L. donovani* strain 1S2D (MHOM/SD/62/1S-CL2D) was obtained from Henry Murray, Weill Cornell Medical College, NY, USA, and maintained by serial passages in hamsters. Anesthetized hamsters were inoculated by intra-cardiac injection of 5×10^7^ amastigotes purified from hamster infected spleens as described previously (Pescher et al., 2011). The weight of the animals was recorded over time, and the animals were euthanized by CO_2_ asphyxiation before they reached the endpoint of infection represented by a 20% loss of body weight. Amastigotes were then recovered from the infected hamster spleens and used for nucleic acid and protein extractions, or differentiated into promastigotes at 26°C in M199 complete medium (M199, 10% FBS, 25 mM HEPES; 100 µM adenine, 2 mM L-glutamine, 10 µg/ml folic acid, 13.7 µM hemin, 4.2 mM NaHCO_3_, 1× RPMI 1640 vitamins, 8 µM 6-biopterin, 100 units penicillin, and 100 µg/ml streptomycin, pH 7.4). Promastigotes, derived from splenic amastigotes, were collected after two in vitro passages for nucleic acid and protein extractions.

### Experimental design

Strains issued from independent experimental evolution assays are identified by number, e.g., amastigote ama1 and promastigote pro1 are the parasites prepared from hamster H1 (see Figure 1A, Figure 1—source data 1 for details). For comparative analyses, DNA, RNA, proteins, and metabolites were extracted from different splenic ama and their matching pro parasites, the latter being collected for extraction during the exponential growth phase.

### Parasite differentiation and lactacystin treatment

Spleen-derived amastigotes were incubated at 2×10^7^/ml and 26°C in the presence or absence of 10 µM of lactacystin (L6785, Sigma) for 3, 6, 18, and 48 hr in M199 complete medium. At each time point, parasites were collected to control for the presence of paraflagellar rod protein 2 (PFR2) as a promastigote differentiation marker. After one in vitro passage, amastigote-derived promastigotes were incubated at a concentration of 2×10^7^ parasites per ml in the presence or absence of 10 µM of lactacystin for 18 hr. Parasite viability was assessed by FACS analysis after 18 hr of treatment by propidium iodide (1 µg/ml) or YO-PRO-1 (0.2 μM) (# Y3603, Invitrogen) staining as readout using untreated and PFA-treated parasites as controls. Parasites were collected from 25 ml of culture after 18 hr of treatment for quantitative, label-free proteomics analyses.

### Microscopy

Differential interference contrast microscopy was applied on hamster-derived amastigotes during in vitro differentiation to promastigotes using an Axioplan 2 imaging microscope using a 63× oil immersion objective, the AxioVision Rel.4.8 software, and an AxioCam MRm camera (Carl Zeiss). Alternatively, microscopic images of amastigotes in the presence or absence of the lactacystin inhibitor were obtained using the EVOS FL microscope (Life Technologies) at a ×20 magnification.

### Western blot analysis

Protein extracts were obtained from parasites after 3, 6, 18, and 48 hr of differentiation from amastigotes to promastigotes in the presence or absence of lactacystin. Extracts were loaded on 4–12% SDS-PAGE (NuPage 4–12% Bis-Tris gel NP0321BOX, Invitrogen), separated by electrophoresis, and stained with Coomassie Blue or transferred onto PVDF membrane. The presence of PFR2 and α-tubulin was revealed using the anti-PFR2 antibody (kindly provided by Philippe Bastin), an anti-α-tubulin antibody (clone B-5-1-2, Sigma), the corresponding HRP-conjugated secondary antibodies (Invitrogen), and the SuperSignal West Pico PLUS Kit (Thermo Scientific). Protein extracts from lactacystin-treated or untreated amastigotes and promastigotes were labeled with Cy5, loaded on 4–12% SDS-PAGE (NuPage 4–12% Bis-Tris gel NP0321BOX, Invitrogen), separated by electrophoresis, and transferred onto PVDF membrane. The presence of ubiquitinated proteins was revealed using the mouse anti-ubiquitin monoclonal antibody P4D1 (Abcam), the HRP-conjugated anti-mouse secondary antibody (Invitrogen), and the ECL Prime western blotting detection reagent (Cytiva). Images were acquired using the ImageQuant 800 (Cytiva).

### Genome sequencing and data analysis

DNA was prepared from splenic amastigotes and promastigotes at exponential culture phase (three biological replicates). Parasites were centrifuged at 1600 × *g* (pro) or 2000 × *g* (ama) for 10 min at room temperature. Approximately 1 to 5×10^8^ parasites were resuspended in 200 µl PBS, and genomic DNA was purified using DNeasy Blood and Tissue Kit from QIAGEN and RNase A, according to the manufacturer’s instructions. DNA concentrations were measured in duplicate by fluorescence using a Molecular Device fluorescence plate reader (Quant-IT kits, Thermo Fisher Scientific). The quality of DNA was controlled by determining the DNA Integrity Number analyzing 20 ng of DNA on a TapeStation 4200 (Agilent). One µg of genomic DNA was used to prepare a library for whole-genome sequencing on an automated platform, using the Illumina ‘TruSeq DNA PCR-Free Library Preparation Kit’, according to the manufacturer’s instructions. After normalization and quality control, qualified libraries were sequenced on a HiSeqX5 platform from Illumina (Illumina Inc, CA, USA) at the Centre National de Recherche en Génétique Humaine (CEA, Evry, France), generating paired-end, 150 bp reads. Sequence quality parameters were assessed throughout the sequencing run. Standard bioinformatics analysis of sequencing data was based on the Illumina pipeline to generate a FASTQ file for each sample.

Genomic DNA reads were aligned to the *L. donovani* Ld1S reference genome (https://www.ncbi.nlm.nih.gov/bioproject/PRJNA396645, GCA_002243465.1) with BWA mem (version 0.7.12) with the flag -M to mark shorter split hits as secondary. Samtools fixmate, sort, and index (version 1.3) were used to process the alignment files and conversion into bam format (Li et al., 2009). RealignerTargetCreator and IndelRealigner from the GATK suite were run to homogenize indels (DePristo et al., 2011). Eventually, PCR and optical duplicates were labeled with Picard MarkDuplicates (version 1.94 (1484)) (https://broadinstitute.github.io/picard/) using the option ‘VALIDATION_STRINGENCY = LENIENT’. For each read alignment file, Samtools view (version 1.3) and BEDTools genomecov (version 2.25.0) were used to measure the sequencing depth of each nucleotide (Quinlan and Hall, 2010). Samtools was run with options ‘-q 50 -F 1028’ to discard reads with a low map quality score or potential duplicates, while BEDTools genomecov was run with options ‘-d -split’ to compute the coverage of each nucleotide. The coverage of each nucleotide was divided by the median genomic coverage. This normalization is done to account for library size differences. The chromosome sequencing coverage was used to evaluate aneuploidy. Then for each sample and for each chromosome, the median sequencing coverage was computed for contiguous windows of 2500 bases. As previously published (Prieto Barja et al., 2017), the stably disomic chromosome 36 was used to normalize chromosome read depth and to estimate chromosome polysomy levels in each sample. Gene counts were produced using featureCounts (version 1.4.6-p3; Liao et al., 2014) with these parameters: -s 0 -t gene -g gene_id and were normalized according to the median-ratio method.

For the generation of the chromosome median somy score heatmap, mean coverage in 300 bp bins as generated by the GIP pipeline (Späth and Bussotti, 2022) were used to compute somy scores per chromosome by first normalizing bin scores for a sample by their median across the entire genome (to obtain comparable values between samples), multiplying by two (to scale somy values to the default diploid state assumed for most of the genome), and then taking the median across bins belonging to a given chromosome (see also here). These chromosome scores were then displayed on a sample × chromosome heatmap, excluding the maxicircle . This was done using the Pandas 1.4.2 (McKinney, 2010; Reback et al., 2021) Pandas-dev/pandas: Pandas 1.2.4 (https://zenodo.org/records/4681666; Reback et al., 2021), Matplotlib 3.5.1 (Hunter, 2007a), and Seaborn 0.11.2 (Waskom, 2021) Python libraries.

For the assessment of gene coverage ratios, normalized mean coverages per gene are reported by GIP (version 1.1.0; Späth and Bussotti, 2022), as the mean coverage of the gene divided by the median coverage of the chromosome containing the gene. For a given gene, and a given amastigote-promastigote pair, a gene coverage ratio was computed by adding 0.1 to the gene coverages of the individual samples, in order to avoid zero division errors. The genes were sorted by genomic coordinate and assigned successive integers to create a ‘genomic index’. Gene coverage ratios were then plotted along this genomic index, using the same Python libraries as above.

For the SNP analysis, alternative (Alt) allele frequencies for SNPs were retrieved from the output of the SNV giptools module (provided along with the GIP pipeline).

### RNA-seq analysis

Total RNA was extracted from splenic amastigotes and promastigotes in exponential culture phase (four biological replicates). Amastigotes and promastigotes were centrifuged respectively at 2000 × *g* or 1600 × *g* for 10 min at 4°C, and resuspended in the lysis buffer supplied with the Nucleospin RNA Plus Kit. The samples were stored at –80°C and RNA extractions were performed according to the manufacturer’s instructions, including a DNase treatment. RNA integrity was validated using the Agilent Bioanalyzer. DNase-treated RNA extracts were used for library preparation. Libraries were constructed using an Illumina Stranded mRNA Prep (Illumina, USA) following the supplier’s recommendations. RNA sequencing was performed at the Biomics Center (Institut Pasteur, Paris, France) on the Illumina NextSeq 2000 platform for a target of 40 M paired-end reads per sample.

The RNA-seq analysis was performed using the Sequana RNA-seq pipeline (version 0.20.1, https://github.com/sequana/sequana_rnaseq; Cokelaer, 2026b) from the Sequana project (Cokelaer et al., 2017). The pipeline workflow manager was Snakemake 7.3.2 (Köster and Rahmann, 2012). The pipeline was executed with default parameters, and bioinformatics software are available as reproducible containers from the Damona project (https://github.com/cokelaer/damona, Cokelaer, 2026a). Reads were mapped to the *L. donovani* reference using bowtie2 2.4.5 (Langmead and Salzberg, 2012). Gene-level quantification was conducted using FeatureCounts 2.0.1 (Liao et al., 2014) assigning reads to genomic features based on the gene annotation and gene_id attribute, while accounting for strand-specificity information. Differential expression analysis was performed using DESeq2 v1.24.0 (Love et al., 2014) scripts available in the Sequana library. Statistical testing identified differentially expressed genes by comparing amastigote and promastigote sample groups, with significance determined using Benjamini-Hochberg adjusted p-values (false discovery rate [FDR]<0.05).

To specifically assess the differential expression of snoRNA, RNA-seq libraries from promastigote and amastigote stages were mapped to the Ld1S genome, and snoRNA read counts were obtained using BEDTools multiBamCov. For snoRNAs with multiple copies, read counts were summed per sample to generate a single expression value per snoRNA family. DESeq2 was then used to perform differential expression analysis between amastigote and promastigote stages based on the summed snoRNA counts.

### Label-free quantitative total proteome analyses

Four biological replicates of untreated amastigotes (ama and ama-18h), promastigotes (pro), and lactacystin-treated parasites (ama-lacta and pro-lacta) (Figure 1, Figure 1—figure supplement 1) were washed three times with cold PBS at 2000 × *g* or 1600 × *g* for 10 min at 4°C. Parasite lysates were prepared in eFASP lysis buffer (4% SDS/0.2% DCA/50 mM TCEP/50 mM ammonium bicarbonate buffer pH 8) (Erde et al., 2014). For detailed protocol, see Appendix 1. MS scans were acquired at a resolution of 70,000 and MS/MS scans (fixed first mass 100 m/z) at a resolution of 17,500. The AGC target and maximum injection time for the survey scans and the MS/MS scans were set to 3×10^6^ for 20 ms and 10^6^ for 60 ms, respectively. An automatic selection of the 10 most intense precursor ions was activated (top 10) with a 40 s dynamic exclusion. The isolation window was set to 1.6 m/z and normalized collision energy fixed to 28 for HCD fragmentation. We used a minimum AGC target of 10^4^ corresponding to an intensity threshold of 1.7×10^5^. Unassigned precursor ion charge states, as well as 1, 7, 8, and >8 charged states, were rejected, and match was disabled.

### Label-free quantitative phosphoproteome analyses

Amastigote (ama) and promastigote (pro) parasites from four biological replicates were washed three times by centrifugation in cold M199 at, respectively, 2000 × *g* or 1600 × *g* for 10 min at 4°C. Samples were incubated for 10 min at 4°C in lysis buffer (1 ml per 1.5×10^9^ parasites) consisting of 8 M urea, 50 mM Tris, supplemented with a protease inhibitor cocktail (cOmplete from Roche) and a phosphatase inhibitor cocktail (PhosStop from Roche). Following sonication for 5 min using a sequence of 10 s pulse and 20 s pause, the lysates were centrifuged for 15 min at 14,000 × *g* and 4°C, and the supernatant was collected and stored at –80°C until use. Proteins were quantified by RC DC protein assay (Bio-Rad) and adjusted to 1.3 µg/µl in lysis buffer. For detailed protocol, see Appendix 1. Phosphopeptide enrichment was carried out as described in Matheron et al., 2014 and detailed Appendix 1. All analyses were performed on a Q Exactive HF Mass Spectrometer (Thermo Fisher Scientific) coupled with a Proxeon EASY-nLC 1000 (Thermo Fisher Scientific) as detailed in Appendix 1. Details for the proteomics and phosphoproteomics analyses following the data acquisition are available in Appendix 1. Results are presented in Figure 4—source data 1 and Figure 5—source data 1.

Phosphopeptides were selected for Gene Ontology (GO) enrichment analyses if (i) they were exclusively identified in one of the two stages, (ii) they showed significant, stage-specific changes in phosphopeptide abundance (fold change≥2, adj. p-value<0.01) even if the corresponding protein was not detected in the total proteome analysis, and (iii) they showed a significant increase in relative phosphorylation normalized to protein abundance (p-value<0.05) as calculated by the ratio ‘change in phosphopeptide abundance’ vs ‘change in protein abundance’ using a cutoff of fold changes≥2 and adj. p-value<0.01 for both analyses (see Figure 5—source data 2).

### Metabolomic analysis

The sample extraction was performed as described previously (Creek et al., 2012). Briefly, 10^8^ cells were used per each 200 μl sample (4 replicates/stage). First, cells were rapidly quenched in a dry ice/ethanol bath to 4°C, then centrifuged, washed with 1× PBS, and resuspended in the extraction solvent (chloroform:methanol:water, 1:3:1). After vigorous shaking at 4°C for 1 hr, extracts were centrifuged (16,000 × *g*, 4°C, 10 min) and the supernatants collected and stored at –80°C until the analysis. LC-MS analyses were performed using separation on 150×4.6 mm, 5 mm ZIC-pHILIC (Merck) on UltiMate 3000 RSLC (Thermo Scientific) followed by mass detection on an Orbitrap Exactive mass spectrometer (Thermo Fisher) at Glasgow Polyomics. Analyses were performed in positive and negative polarity switching mode, using 10 μl injection volume and a flow rate of 300 μl/min over 26 min on the column maintained at 30°C, as follows: 0–20 min 20–80% solution A, 15–17 min 95% solution A, 17–26 min 20% solution A where solution A is 20 mM ammonium carbonate in water and solution B is acetonitrile. The samples were run alongside 249 authentic standards at 10 μM each. Mass spectrometry data was processed using Mzmatch (Scheltema et al., 2011) and Ideom (Creek et al., 2012) software. Unique signals were extracted using the centwave algorithm and matched across biological replicates based on mass-to-charge ratio and retention time. These grouped peaks were then filtered based on relative standard deviation and combined into a single file. The combined sets were then filtered on signal-to-noise score, minimum intensity, and minimum detections. The final peak set was then gap-filled and converted to text for use. Putative metabolite identification corresponds to the most part to Metabolite Standards Initiative (MSI) level 2 (mass only, and thus only considered a tentative annotation), whereas metabolites matching in retention time to an included standard correspond to level 1 (considered likely an accurate annotation). Peaks having an area with root squared deviation across pooled samples>50% were excluded, as were those with a retention time<4 min (due to poor resolution).

### Localizing rRNA modification on *Leishmania* cryo-EM ribosome structure

The cryo-EM atomic model of *Leishmania major* 80S ribosomes bound to mRNA and all three tRNAs (PDB: 8RXH) was used to project rRNA modifications of the ribosome. Figures were generated using UCSF Chimera-X software (Pettersen et al., 2021).

### Systems-level analyses

A list of *L. donovani* GO terms (Bussotti et al., 2021) was built in-house. The Biological Networks Gene Ontology tool (BiNGO) plugin of the Cytoscape software package (version 3.8.2) was used to map and visualize functional enrichments in each dataset based on the GO hierarchy. A Benjamini-Hochberg FDR with a significance level of 0.05 was applied. Cluster efficiency represents the percentage of genes for a given GO term compared to the total number of genes with any GO annotation in the considered set of genes. Enrichment score corresponds to the percentage of genes for a given GO term compared to all the genes sharing the same GO term in the genome. Word Cloud of GO enrichment analysis limited to GO Slim terms and a threshold of p-value<0.05 for the category Biological Process was performed on TriTrypdb (https://tritrypdb.org) using the *L. donovani* LdBPK orthologs (Supplementary file 1) for all genes that showed statistically significant expression changes (p-value <0.01) at both RNA or protein levels.

For the phosphoproteomic work, phosphopeptides were selected for GO enrichment analyses if (i) they were exclusively identified in one of the two stages, (ii) they showed significant, stage-specific changes in phosphopeptide abundance (fold change≥2, adj. p-value<0.01) even if the corresponding protein was not detected in the total proteome analysis, and (iii) they showed a significant increase in relative phosphorylation normalized to protein abundance (p-value<0.05) as calculated by the ratio ‘change in phosphopeptide abundance’ vs ‘change in protein abundance’ using a cutoff of fold changes≥2 and adj. p-value<0.01 for both analyses (Figure 5—source data 3).

Functional enrichment networks were built with the STRING plugin of the Cytoscape software package (version 3.8.2) using the *L. infantum* orthologs (Supplementary file 1), and the full STRING network with a confidence score cutoff of 0.4. An FDR with a significance level of 0.05 was applied. The ‘ClusterProfiler’ package (version 4.2.2) of R was used for KEGG (Kyoto Encyclopedia of Genes and Genomes) gene set enrichment analysis and for data mapping on metabolic pathways available in the KEGG database. Results were visualized using the ‘pathview’ packages (version 1.34.0).

## Data Availability

The mass spectrometry proteomics data were deposited to the ProteomeXchange Consortium via the PRIDE partner repository with the dataset identifiers PXD035697 (phosphoproteome data) and PXD035698 (proteome upon lactacystin treatment) (Perez-Riverol et al., 2019). The DNAseq data have been deposited in NCBI's Gene Expression Omnibus (Edgar et al., 2002) and are accessible in the BioProject PRJNA1231373 (https://www.ncbi.nlm.nih.gov/bioproject/PRJNA1231373). The RNAseq data have been deposited in ArrayExpress (EMBL-EBI) (Athar et al., 2019) under the accession number E-MTAB-16528. The mass spectrometry metabolomic data are available on Figshare at https://doi.org/10.6084/m9.figshare.31027849. The scripts for GIP and giptools are available at https://github.com/susefranssen/Global_genome_diversity_Ldonovani_complex (Franssen, 2020). Figure 1—source data 1: DNA - Somy score DNA - Gene copy number variation (gCNV) Figure 1—source data 2: DNA - Gene copy number variation (gCNV) Figure 1—source data 3: DNA - frequency of SNV Figure 2—source data 1: RNA - Transcriptomic analysis ama vs pro Figure 2—source data 2: RNA - snoRNAs Figure 3—source data 1: Protein - Proteomic analysis ama vs pro Figure 3—source data 2: RNAxProtein - Post-transcriptional regulation of gene expression Figure 4—source data 1: Protein - Global proteomic analysis of lactacystin treated ama and pro vs untreated parasites Figure 4—source data 2: Protein - Proteins modulated during the first 18h of ama to pro differentiation Figure 4—source data 3: Protein - Protein stabilization in ama and pro in presence of lactacystine Figure 5—source data 1: Phosphoprotein - Phosphoproteomic analysis of ama vs pro Figure 5—source data 2: Phosphoprotein - Stage-specific relative phosphorylation changes Figure 5—source data 3: Phosphoprotein - Stage-specific functional networks (STRING) Figure 6—source data 1: Phosphoprotein - Stage-specific interaction networks for selected phosphoproteins (STRING) Figure 4—source data 4: PDF file containing original western blot and SDS-PAGE for Figure 4C, indicating the relevant bands and treatments Figure 4—source data 5: Source images of the western blot and SDS-PAGE presented in Figure 4C. Figure 4—figure supplement 3—source data 1: PDF file containing original western blots presented in Figure 4—figure supplement 3C indicating the relevant bands and treatments Figure 4—figure supplement 3—source data 2: Source images of the western blots presented in Figure 4—figure supplement 3C. Figure 4—figure supplement 3—source data 1: PDF file containing original western blots presented in Figure 4—figure supplement 3D indicating the relevant bands and treatments Figure 4—figure supplement 3—source data 2: Source images of the western blots presented in Figure 4—figure supplement 3D. The following datasets were generated: SpaethG 2022Five-layer systems analysis of Leishmania stage differentiation reveals an essential role for protein degradation in parasite developmentPRIDEPXD03569710.7554/eLife.111115PMC1346654442583824 SpaethG 2022Five-layer systems analysis of Leishmania stage differentiation reveals an essential role for protein degradation in parasite developmentPRIDEPXD03569810.7554/eLife.111115PMC1346654442583824 SpaethG 2025ImmunoLeish: genomic adaptation of *L. donovani* Ld1S parasites during rodent infectionNCBI BioProjectPRJNA1231373 CokelaerT PescherP SpaethG 2026Stage-specific analysis of *L. donovani* transcriptomeArray ExpressE-MTAB-16528 KovarovaJ 2026Metabolomics dataset promastigotes vs. splenic amastigotesfigshare10.6084/m9.figshare.31027849

## Additional files

Supplementary file 1. Table for Ld1S, LdBPK, and *L. infantum* gene/protein IDs equivalence.

MDAR checklist

## Appendix 1

### Supplement for materials and methods

#### Label-free quantitative total proteome analyses

Untreated amastigotes (ama and ama-18h) and promastigotes (pro), and lactacystin-treated parasites (ama-lacta and pro-lacta) were recovered and washed three times with cold PBS at 2000 × *g* or 1600 × *g* for 10 min at 4°C. Parasite lysates were prepared in eFASP lysis buffer (4% SDS/0.2% DCA/50 mM TCEP/50 mM ammonium bicarbonate buffer pH 8) (Erde et al., 2014). Filter units and collection tubes were incubated overnight in 5% (vol/vol) Tween-20. All buffer exchanges were carried out by centrifugation at 14,000 × *g* for 10 min. For each sample, 50 µg of proteins were transferred into 30,000 Da MWCO centrifugal units (Amicon Centrifugal Filters, Merck) adjusted to 250 μl with exchange buffer (8 M urea, 0.2% DCA, 100 mM ammonium bicarbonate, pH 8) and washed three times with 200 μl exchange buffer, after which samples were incubated in alkylation buffer (50 mM chloroacetamide, urea 8 M, 100 mM ammonium bicarbonate, pH 8) in the dark for 1 hr. The alkylating agent was then replaced with 200 μl exchange buffer, followed by three washes in 200 μl digestion buffer (0.2% DCA/50 mM ammonium bicarbonate buffer, pH 8). Digestion was performed at 37°C overnight in 100 μl with Sequencing Grade Modified Trypsin (Promega – V5111) at a protein:trypsin ratio of 50:1. Two rounds of 50 μl of 50 mM ammonium bicarbonate pH 8 were used to recover the peptide-rich solution by centrifugation. DCA was removed by acidification and phase transfer. Finally, peptides were speed-vacuum dried and resuspended in 2% acetonitrile (ACN), 0.1% formic acid (FA) prior to LC-MS/MS analysis.

Tryptic peptides from eFASP digestion were analyzed on a Q Exactive Plus instrument (Thermo Fisher Scientific) coupled with an EASY nLC 1200 chromatography system (Thermo Fisher Scientific). Samples were loaded into a home-made 44 cm C18 column (1.9 μm particles, 100 Å pore size, ReproSil-Pur Basic C18, Dr. Maisch GmbH, Ammerbuch-Entringen, Germany). Column equilibration and peptide loading were performed at 900 bars in buffer A (0.1% FA). Peptides were eluted at a flow rate of 250 nl/min over 182 min with a multi-step gradient from 2% to 7% buffer B (80% ACN, 0.1% FA) during 5 min, 7% to 23% buffer B for 130 min, 23% to 45% buffer B for 20 min, 45% to 95% buffer B for 5 min. Column temperature was set to 60°C. MS data were acquired using Xcalibur software using a data-dependent method. MS scans were acquired at a resolution of 70,000 and MS/MS scans (fixed first mass 100 m/z) at a resolution of 17,500. The AGC target and maximum injection time for the survey scans and the MS/MS scans were set to 3×10^6^ for 20 ms and 10^6^ for 60 ms, respectively. An automatic selection of the 10 most intense precursor ions was activated (top 10) with a 40 s dynamic exclusion. The isolation window was set to 1.6 m/z and normalized collision energy fixed to 28 for HCD fragmentation. We used a minimum AGC target of 10^4^ corresponding to an intensity threshold of 1.7×10^5^. Unassigned precursor ion charge states as well as 1, 7, 8, and >8 charged states were rejected, and peptide match was disabled.

#### Label-free quantitative phosphoproteome analyses

Amastigote (ama) and promastigote (pro) parasites were recovered and washed three times by centrifugation in cold M199 at, respectively, 2000 × *g* or 1600 × *g* for 10 min at 4°C. Samples were incubated for 10 min at 4°C in lysis buffer (1 ml per 1.5×10^9^ promastigotes) consisting of 8 M urea, 50 mM Tris, supplemented with a protease inhibitor cocktail (cOmplete from Roche) and a phosphatase inhibitor cocktail (PhosStop from Roche). Following sonication for 5 min using a sequence of 10 s pulse and 20 s pause, the lysates were centrifuged for 15 min at 14,000 × *g* and 4°C, and the supernatant was collected and stored at –80°C until use. Proteins were quantified by RC DC protein assay (Bio-Rad) and adjusted to 1.3 µg/µl in lysis buffer. Disulfide bridges were reduced in 5 mM DTT (Sigma – 43815) for 30 min and alkylated in 20 mM iodoacetamide (Sigma – I1149) for 30 min at room temperature in the dark. Protein samples were diluted 10-fold in 50 mM Tris-HCl and digested with Sequencing Grade Modified Trypsin (Promega – V5111) at a protein:trypsin ratio of 50:1 overnight. Then, a second digestion was performed to complete this step. Proteolysis was stopped by adding formic acid (FA, Fluka – 94318) at a 1% final concentration. Resulting peptides were desalted using Sep-Pak SPE cartridge (Waters) according to the manufacturer’s instructions. Peptides were concentrated to almost dryness and were resuspended in 2% ACN/0.1% FA just before LC-MS/MS injection.

Phosphopeptide enrichment was carried out as described in Matheron et al., 2014. A slurry of 10 mg/ml of the TiO_2_ beads (Sachtopore, Sachtleben Chemie, Germany) was prepared in TiO_2_ buffer (30% ACN/0.1% trifluoroacetic acid [TFA]). For each sample, GELoader tip was prepared in a Stage-Tips manner using a C8 plug previously activated in methanol and washed with TiO_2_ buffer. Then, 40 μl of the slurry was packed by centrifugation (100 × *g*) in GELoader tips. The microcolumns were conditioned with 50 μl of loading buffer (80% ACN, 6% TFA, 40 mg/ml glycolic acid) by centrifugation (150 × *g*). The samples were resuspended in loading buffer at a concentration of 2.5 µg/µl and 250 µg were loaded on the microcolumns by centrifugation (100 × *g*). The microcolumns were washed with 50 μl of 80% ACN/6% TFA and then 100 μl of 50% ACN/0.1% TFA by centrifugation (150 × *g*). Phosphopeptides were eluted into 60 μl of 10% NH_4_OH and then into 5 μl of 80% ACN/2% FA by centrifugation (100 × *g*). 3.5 μl of 100% FA was added for further acidification.

All analyses were performed on a Q Exactive HF Mass Spectrometer (Thermo Fisher Scientific) coupled with a Proxeon EASY-nLC 1000 (Thermo Fisher Scientific). Phosphopeptides were injected into a home-made 50 cm C18 column (1.9 μm particles, 100 Å pore size, ReproSil-Pur Basic C18, Dr. Maisch GmbH, Ammerbuch-Entringen, Germany). Column equilibration and peptide loading were performed at 250 nl/min in buffer A (0.1% FA). Phosphopeptides were separated at a flow rate of 250 nL/min over 202 min with a multi-step gradient of 2–10% buffer B (80% ACN, 0.1% FA) for 40 min, 10–30% buffer B for 110 min, 30–60% buffer B for 20 min, and 60–80% buffer B for 1 min. Column temperature was set to 60°C. MS data were acquired using Xcalibur software using a data-dependent method. MS scans were acquired at a resolution of 60,000 and MS/MS scans (fixed first mass 100 m/z) at a resolution of 15,000. The AGC target and maximum injection time for the survey scans and the MS/MS scans were set to 3×10^6^ for 20 ms and 10^6^ for 60 ms, respectively. An automatic selection of the 10 most intense precursor ions was activated (top 10) with a 40 s dynamic exclusion. The isolation window was set to 1.6 m/z and normalized collision energy fixed to 27 for HCD fragmentation. We used an underfill ratio of 1% corresponding to an intensity threshold of 1.7E^5^. No charged states were rejected, and peptide match was preferred.

#### Proteomics and phosphoproteomics analyses

Raw data were analyzed using MaxQuant software (version 1.5.3.8) (Cox and Mann, 2008) using the Andromeda search engine (Cox et al., 2011). The MS/MS spectra were searched against the Ld1S database (https://www.ncbi.nlm.nih.gov/bioproject/PRJNA396645, GCA_002243465.1). The settings for the search included (i) trypsin digestion with a maximum of two missed cleavages, (ii) variable modifications for methionine oxidation, N-terminal acetylation, and lysine ubiquitinylation, and (iii) fixed modification for cysteine carbamidomethylation. For phosphorylation event investigation, serine, threonine, and tyrosine phosphorylation were added as variable modifications. The minimum peptide length was set to seven amino acids, and the FDR for peptide and protein identification was set to 0.01. The main search peptide tolerance was set to 4.5 ppm and to 20 ppm for the MS/MS match tolerance. The setting ‘second peptides’ was enabled to identify co-fragmentation events. Quantification was performed using the XIC-based label-free quantification (LFQ) algorithm with the Fast LFQ mode as previously described (Cox et al., 2014). Unique and razor peptides, including modified peptides, with at least two ratio counts were accepted for quantification.

The workflows applied for the proteomics and phosphoproteomics data analysis are comparable to those previously used in this context (Garcia-Garcia et al., 2022).

For the differential analyses of proteome data, proteins categorized as ‘reverse’, ‘contaminant’, and ‘only identified by site’ were discarded from the list of identified proteins. After log_2_ transformation, LFQ values were normalized by median centering within conditions (*normalizeD* function of the R package *DAPAR;* Wieczorek et al., 2017). Remaining proteins without any LFQ value in one of the conditions (either ama or pro) and at least two values in the other condition were considered as exclusively expressed proteins. Missing values across the four biological replicates were imputed using the imp.norm function of the R package norm (norm: Analysis of multivariate normal datasets with missing values. 2013 R package, version 1.0–9.5). A limma t-test was applied to determine proteins with a significant difference in abundance while imposing a minimal fold change of 2 between the conditions to conclude that they are differentially abundant (Ritchie et al., 2015; Smyth, 2005). An adaptive Benjamini-Hochberg procedure was applied on the resulting p-values using the function *adjust.p* of the R package *cp4p* (Giai Gianetto et al., 2016) and the robust method described in Pounds and Cheng, 2006, to estimate the proportion of true null hypotheses among the set of statistical tests. The proteins associated with an adjusted p-value inferior to an FDR of 0.01 have been considered as significant and differentially abundant proteins. The volcano plot is shown in Figure 2B and contains on their side the iBAQ (Scheltema et al., 2011) values of proteins present or absent in one of the two conditions compared.

The workflow of the phosphoproteome analysis is close to the proteomics data analysis with some minor adaptations (Giai Gianetto, 2023). Phosphosites were only considered if detected with high confidence (identification FDR<1%) and high localization confidence (localization probability>0.75) in at least one replicate per condition. This criterion was chosen to retain biologically relevant, low-abundance phosphosites, which are more difficult to identify and are often stochastically sampled in phosphoproteomics datasets. Intensities of proteins and phosphopeptides were normalized by condition using a median-centering function from DAPAR (Barbiero et al., 2024), and their missing values were imputed using the impute.mle function of the imp4p R package (Gianetto et al., 2020) when at least one observed value was present in that condition. This algorithm imputes values in a condition only when an intensity value has been quantified in at least one of the samples of the considered condition. The quantification profiles (modified peptide and parent unmodified protein quantified/not quantified in at least one sample of a condition) can be viewed in the ‘Absent/Present’ columns of Figure 5—source data 1. For the differential analysis of one condition vs another one, two statistical tests were used. First, a moderated t-test was performed, thanks to the limma R package (Ritchie et al., 2015; Smyth, 2005) to determine whether a phosphorylated peptide is significantly differentially abundant between both conditions. Moreover, phosphorylated peptides quantified in one condition and not in the other were also considered differentially abundant. We applied a contrasted t-test to compare the variation in abundance of each modified peptide to the one of its parent unmodified protein using the limma R package (Ritchie et al., 2015; Smyth, 2005). This second test allows the calculation of a normalized phosphorylation change defined as the ratio of the change in phosphopeptide abundance relative to the change in the corresponding total protein abundance for each condition (paragraph 3.9 in Giai Gianetto, 2023). An adaptive Benjamini-Hochberg procedure was applied on the resulting p-values, thanks to the adjust.p function of the R package cp4p (Giai Gianetto et al., 2016) using the Pounds and Cheng, 2006 (McConville and Ralton, 1997) method to control the FDR level. The phosphorylated peptides associated with an adjusted p-value inferior to an FDR of 1% have been considered as significantly and differentially abundant between the compared conditions. Note that this second test can be performed only when there are quantified intensity values for both the non-modified peptide and its parent protein. Interesting cases also emerge from the absence of quantified values for the belonging protein. Consequently, differentially abundant peptides that are associated with proteins from which no fold change can be computed were also considered in the final list of differentially abundant peptides that evolve differently from their parent protein. Results of these differential analyses are summarized in Figure 5A and available in Figure 5—source data 1.
