# Supplementary material for: Five-layer systems analysis of Leishmania stage differentiation reveals an essential role for protein degradation in parasite development
Source: eLife. 2026 Aug 12;15:RP111115. doi: 10.7554/eLife.111115 (PMC13466544; doi:10.7554/eLife.111115)

Figure 4-figure supplement 3D

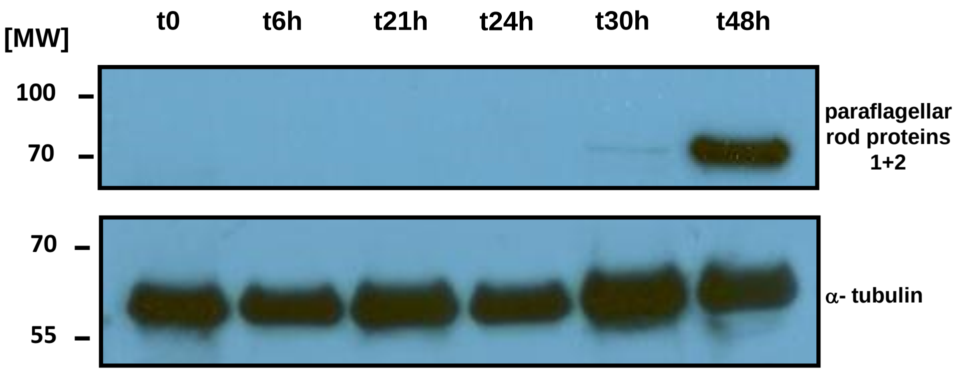

Figure 4-figure supplement 3D - uncropped

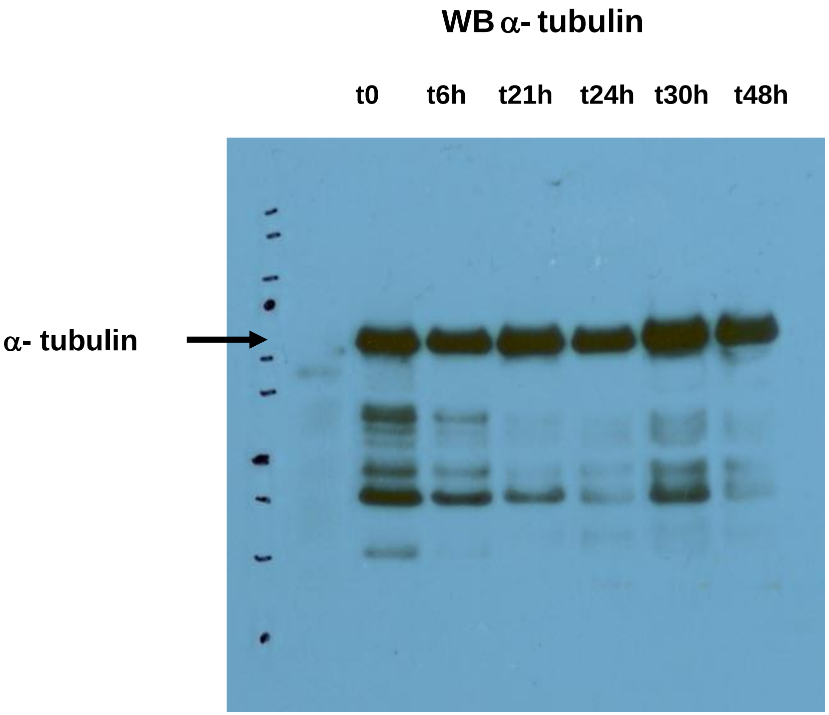

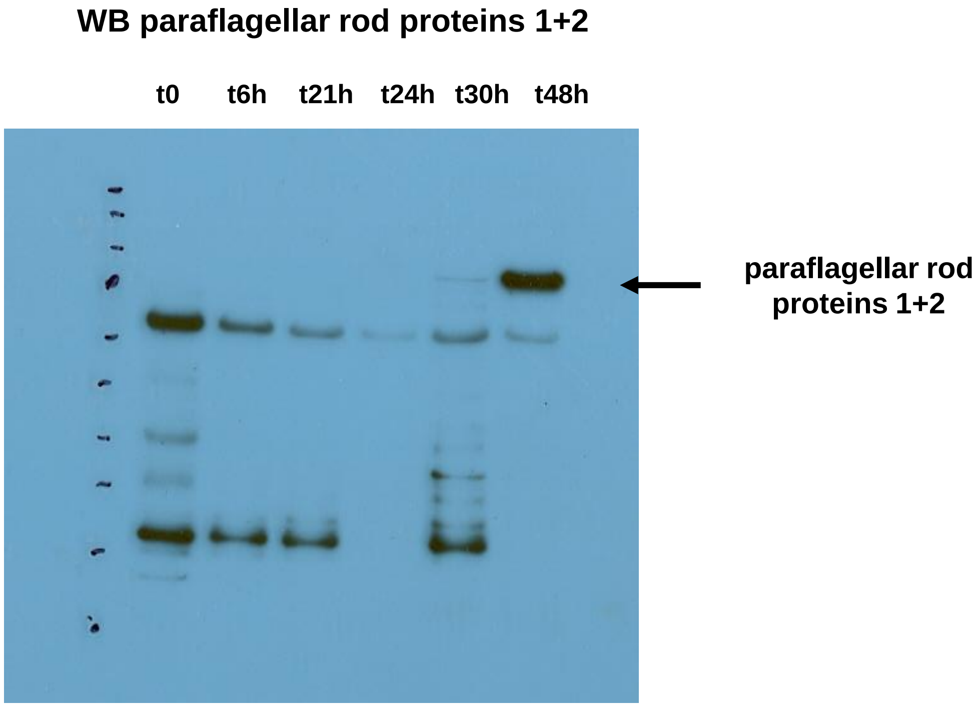

Supplement: Supplementary file 4 [file elife-111115-fig4-figsupp3-data1.zip › Figure 4-figure supplement 3D-source Data 19.pdf]
