# Supplementary material for: Five-layer systems analysis of Leishmania stage differentiation reveals an essential role for protein degradation in parasite development
Source: eLife. 2026 Aug 12;15:RP111115. doi: 10.7554/eLife.111115 (PMC13466544; doi:10.7554/eLife.111115)
Supplement: Peer review history

# Editor report: eLife Assessment

**DOI:** 10.7554/eLife.111115.3.sa0

**Authors:** Dominique Soldati-Favre

**Affiliations:** University of Geneva Switzerland

**Keywords:** Convincing, Important

This **important** study provides a comprehensive multi-omics characterization of *Leishmania donovani* stage differentiation, offering insights into the molecular basis of parasite adaptation across host environments. The authors present **convincing** evidence that stage transitions are not driven by genomic variation but instead rely on coordinated post-transcriptional regulation, including mRNA turnover, translation, and protein degradation. Although experimental validation of these findings and conclusions remains to be completed, the integration of diverse, high-quality datasets establishes a robust resource that will be of broad utility to researchers investigating *Leishmania* biology and life-cycle progression.

[Editors' note: this paper was reviewed by Review Commons.]

# Referee report: Reviewer #1 (Public review)

**DOI:** 10.7554/eLife.111115.3.sa1

[Editors' note: this version has been assessed by the Reviewing Editor without further input from the original reviewers. The authors have addressed the comments raised in the previous round of review.]

Summary:

The authors describe co-regulated gene modules underlying stage differentiation in Leishmania donovani through a system-level analysis of multiple molecular layers. Using amastigotes isolated from infected hamster spleens and corresponding culture-derived promastigotes, they analyzed genomic variation, transcript abundance, protein levels, phosphorylation states, and metabolite profiles. By combining these, the study identified potential regulatory mechanisms associated with parasite differentiation and generated hypotheses regarding how gene expression is coordinated across different levels.

Strengths:

A major strength of the study is the breadth of the dataset generated. The integration provides an unusually comprehensive view of molecular changes associated with Leishmania differentiation in vitro. Such multi-layer datasets involving bona fide vertebrate host stages remain relatively rare in parasitology and will likely become a valuable resource for the molecular parasitology community. In addition, the use of amastigotes isolated from infected hamsters rather than relying on axenic models provided a biologically relevant framework for the analyses.

The revised manuscript improved several aspects of the original. The RNA-seq analysis is described with a clearer pipeline, and several claims regarding causal regulatory feedback associations have been appropriately toned down. Among the observations reported, the association between parasite differentiation and proteasome-mediated protein degradation is particularly remarkable. The combination of quantitative proteomics with pharmacological inhibition of the proteasome with lactacystin provides support for a role for protein turnover in developmental transitions and paves the way for future mechanistic studies.

Weaknesses:

Most regulatory interpretations remain largely inferential or indirect. The integration identifies correlations between different levels, but direct functional validation is limited/absent. Many of the descriptions should not be interpreted as validated. As highlighted by the authors in this revised version, the mechanistic studies will be part of future work and are beyond the scope of the current work. Of note, the attempt to confirm lactacystin-induced inhibition of proteasomal activity via anti-polyUb immunoblotting did not demonstrate the expected outcome of increase in overall poly-ubiquitylation.Editors' note: this version has been assessed by the Reviewing Editor without further input from the original reviewers. The authors have addressed the comments raised in the previous round of review.

# Referee report: Reviewer #2 (Public review)

**DOI:** 10.7554/eLife.111115.3.sa2

Pescher and colleagues present a revised manuscript detailing the multi-omic characterisation of Leishmania donovani amastigote to promastigote differentiation and integration of this data. The molecular pathways that regulate Leishmania life-stage transitions are still poorly understood, with many approaches exploring single proteins/RNAs etc in a reductionist manner. This paper takes a systems-scale approach and does a good job of integrating the disparate -omics datasets to generate hypotheses about the intersections of regulatory proteins that are associated with life-cycle progression. The differentiation step studied is from amastigote to promastigote using hamster-derived amastigotes which is a major strength. The use of hamsters permits the extraction of parasites that are host adapted and represent "normal", host-adapted Leishmania ploidy; the promastigote experiments are performed at a low passage number. Therefore, this is a strength or the work as it reduces the interference from the biological plasticity of Leishmania when it is cultured outside the host for prolonged periods. The multi-omics datasets presented are robust in their acquisition and analysis and will form an excellent resource for researchers studying the molecular events (particularly proteasomal protein degradation, and phosphorylation) during life-stage progression.

Overall, in the absence of follow up experiments on specific individual examples, some of the claims in the original submission were toned down and reflect a more neutral description of the data now. Significantly, the data still underpin a key role for regulation of the ribosome between the amastigote and promastigote stages (and during the differentiation process). The recursive and reciprocal links between the phosphorylation and ubiquitination systems are interesting and present many opportunities for future investigation.

# Referee report: Reviewer #3 (Public review)

**DOI:** 10.7554/eLife.111115.3.sa3

Summary:

The authors proposed to use 5-layer systems level analysis (genomics, transcriptomics, proteomics / protein degradation, metabolomics, phosphoproteomics) to uncover how post-transcriptional mechanisms regulate stage differentiation in Leishmania donovani.

This enabled the identification of several potential regulatory networks, including the regulation of stage-specific gene clusters by RNA stabilisation or decay, proteasomal degradation and protein phosphorylation.

In the new version of this manuscript, the authors have addressed all questions raised by the reviewers.

Strengths:

Although some observations in this study have already been described in the literature, the integrated analysis applied here provides a novel view on how different levels of post-transcriptional networks regulate Leishmania differentiation. This "5-layer system" represents the first analysis of this depth in kinetoplastid parasites.

The revised version with an increased sample number for the RNA-seq now made the authors assumptions adequate to their obtained data.

The use of a proteasomal inhibitor adds an interesting insight in how protein degradation is involved in the parasite differentiation, confirming previous observations in the literature, and help to explain the discrepancies between mRNA and protein expression in the different stages.

Weaknesses:

While this work provides an impressive and foundational dataset, it opens the door for future research to rigorously validate these initial findings and conclusions.

Significance and Impact in the field.

The different datasets generated in this study will be of great interest to the parasitology community, either to be used for hypothesis generation, to validate data from other sources, etc.

The multi-layered analysis performed here identified a series of potential feedback loops and regulatory networks to be further explored in organisms that lack transcriptional control.

# Author comment: Author response

**DOI:** 10.7554/eLife.111115.3.sa4

**Authors:** Pascale Pescher, Thibaut Douché, Quentin Giai Gianetto, Karen Druart, Julie Kovářová, Blaise Li, Thomas Cokelaer, K Shanmugha Rajan, Laura Piel, Céline Besse, Anne Boland, Jean-François Deleuze, Mariette Matondo, Michael P Barrett, Shulamit Michaeli, Gerald Spaeth

**Affiliations:** Institut Pasteur Paris France; Institut Pasteur Paris France; Institut Pasteur Paris France; Institut Pasteur Paris France; Institute of Parasitology České Budějovice Czech Republic; Institut Pasteur Paris France; Institut Pasteur Paris France; Bar Ilan University Ramat Gan Israel; Institut Pasteur Paris France; Centre National de Recherche en Génomique Humaine Paris France; CNRGH: Commissariat a l'energie atomique et aux energies alternatives Centre National de Recherche en Genomique humaine Paris France; Centre d'Etude du Polymorphisme Humain Paris France; Institut Pasteur, CNRS UMR6047 Paris France; University of Glasgow Glasgow United Kingdom; Bar Ilan University Ramat Gan Israel; Institut Pasteur Paris France

The following is the authors’ response to the original reviews

**Public Reviews:**

**Reviewer #1 (Public review):**

Comments on revised version:

The authors have appropriately addressed my comments and questions from the initial review process. My remaining concern relates to the lack of evidence to confirm proteasomal inhibition by lactacystin in both promastigotes and amastigotes. The immunoblotting experiment newly presented does not reveal a clear increase in the levels of poly-ubiquitylated proteins in treated parasites. In fact, poly-Ub levels were lower at both the 4h and 18h timepoints of treatment. If alternative antibodies or additional immunoblots are not available, the manuscript would benefit from an expanded discussion of this observation and potential explanations. In particular, the interpretation that lactacystin stabilizes ama- and pro-specific degradation would be greatly strengthened by such validation.

**Reviewer #2 (Public review):**

General comments on the revisions:

My view is that the authors have made significant, satisfactory changes that address the comments and queries I made on the original manuscript (Review Commons).

There are two areas where the authors had to make major changes/justifications where further comment is merited, these were:

RNA-seq.

The most significant issue was the originally underpowered RNA-seq which had only two replicates. This has been repeated with four replicates now. This has not led to changes in the interpretation of the data between the original study and this one. One comment that the authors make in the response to this was : "Given the robustness of the stage-specific transcriptome, and the legal constrains associated with the use of animals, we chose to limit the number of replicates to the necessary". Ensuring that animal experiments are properly powered and that maximum robustness of the data from the minimum sample size is an important part of experimental design for ethical use of animal models. Essentially the replication here could have been avoided if the original study had used 1 more animal. However, the new version of RNA-seq brings appropriate confidence to the interpretation of the data.

Phosphoproteomics.

The authors provide a robust justification of their strategy for the phosphoproteomics and highlight the inclusion criteria for phosphosites: "Phosphosites were only considered if detected with high confidence (identification FDR<1%) and high localisation confidence (localisation probability >0.75) in at least one replicate". The way missing values were dealt with is explained "For statistical analyses, missing values within a given condition were imputed with a well-established algorithm (MLE) only when at least one observed value was present in that condition." This fills in some of the gaps I was missing from the original manuscript, and I am satisfied that the data analysis is entirely appropriate for a discovery/system -based approach such as this one. The authors also edit the manuscript to reflect that "occupancy" or "stoichiometry" might not be the best description of what they were presenting and switched to the terminology of "normalised phosphorylation level" - I think this is an appropriate response.

Overall, in the absence of follow up experiments on specific individual examples, some of the claims in the original submission were toned down and reflect a more neutral description of the data now. Significantly, the data still underpin a key role for regulation of the ribosome between the amastigote and promastigote stages (and during the differentiation process). The recursive and reciprocal links between the phosphorylation and ubiquitination systems are interesting and present many opportunities for future investigation.

**Reviewer #3 (Public review):**

Summary:

The authors proposed to use 5-layer systems level analysis (genomics, transcriptomics, proteomics / protein degradation, metabolomics, phosphoproteomics) to uncover how post-transcriptional mechanisms regulate stage differentiation in Leishmania donovani.

This enabled the identification of several potential regulatory networks, including the regulation of stage-specific gene clusters by RNA stabilisation or decay, proteasomal degradation and protein phosphorylation.

In the new version of this manuscript, the authors have addressed all questions raised by the reviewers.

Strengths:

Although some observations in this study have already been described in the literature, the integrated analysis applied here provides a novel view on how different levels of post-transcriptional networks regulate Leishmania differentiation. This "5-layer system" represents the first analysis of this depth in kinetoplastid parasites.

The revised version with an increased sample number for the RNA-seq now made the authors assumptions adequate to their obtained data.

The use of a proteasomal inhibitor adds an interesting insight in how protein degradation is involved in the parasite differentiation, confirming previous observations in the literature, and help to explain the discrepancies between mRNA and protein expression in the different stages.

Weaknesses:

While this work provides an impressive and foundational dataset, it opens the door for future research to rigorously validate these initial findings and conclusions.

Significance and Impact in the field.

The different datasets generated in this study will be of great interest to the parasitology community, either to be used for hypothesis generation, to validate data from other sources, etc.

The multi-layered analysis performed here identified a series of potential feedback loops and regulatory networks to be further explored in organisms that lack transcriptional control.

According to the reviewers’ comments, we made the following minor changes:

As suggested by reviewer 1, we have extended the discussion of the results related to the analysis of the ubiquitination pattern by Western blot analysis as follows: “Proteasome inhibition blocked amastigote-to-promastigote differentiation, without inducing rapid global accumulation of ubiquitinated proteins (Figure S7C, upper panel) consistent with a quiescent-like state and low basal ubiquitin–proteasome system activity in amastigotes. After 18 h, ubiquitination levels remained similar to untreated cells, indicating that protein turnover and ubiquitin accumulation are primarily driven by developmental remodeling rather than acute proteasome inhibition. In promastigotes, the lack of detectable change (Fig. S7C, lower panel) may also reflect high basal ubiquitination, engagement of compensatory pathways such as autophagy, and/or only partial proteasome inhibition.”

**Recommendations for the authors:**

**Reviewer #3 (Recommendations for the authors):**

Minor comments:

- Supplementary figure 3 is not referenced in the main text.

- The authors removed the "infinite" sign from figures 3 and 4 to better present the data according to their chosen approach to missing values when LFQ=0. However, the sign is still present in the respective figure legends, please adjust.

Supplementary Figure 3 (Figure S3) is now referenced in the main text as requested.

The "infinite" sign has been removed from the legends of Figures 3 and 4 as requested.
